# Four decades of climatic fluctuations and fish recruitment stability across a marine‐freshwater gradient

**DOI:** 10.1111/gcb.16266

**Published:** 2022-06-16

**Authors:** Denise D. Colombano, Stephanie M. Carlson, James A. Hobbs, Albert Ruhi

**Affiliations:** ^1^ Department of Environmental Science, Policy, and Management University of California Berkeley Berkeley California USA; ^2^ Region 3 Bay‐Delta Stockton IEP Office California Department of Fish and Wildlife Stockton California USA

**Keywords:** biocomplexity, biological insurance, drought, fisheries, hydroclimate, marine heatwave, nursery, portfolio effect, state‐space models

## Abstract

Investigating the effects of climatic variability on biological diversity, productivity, and stability is key to understanding possible futures for ecosystems under accelerating climate change. A critical question for estuarine ecosystems is, how does climatic variability influence juvenile recruitment of different fish species and life histories that use estuaries as nurseries? Here we examined spatiotemporal abundance trends and environmental responses of 18 fish species that frequently spend the juvenile stage rearing in the San Francisco Estuary, CA, USA. First, we constructed multivariate autoregressive state‐space models using age‐0 fish abundance, freshwater flow (*flow*), and sea surface temperature data (*SST*) collected over four decades. Next, we calculated coefficients of variation (CV) to assess portfolio effects (1) within and among species, life histories (anadromous, marine opportunist, or estuarine dependent), and the whole community; and (2) within and among regions of the estuary. We found that species abundances varied over space and time (increasing, decreasing, or dynamically stable); and in 83% of cases, in response to environmental conditions (wet/dry, cool/warm periods). Anadromous species responded strongly to flow in the upper estuary, marine opportunist species responded to flow and/or SST in the lower estuary, and estuarine dependent species had diverse responses across the estuary. Overall, the whole community when considered across the entire estuary had the lowest CV, and life histories and species provided strong biological insurance to the portfolio (2.4‐ to 3.5‐fold increases in stability, respectively). Spatial insurance also increased stability, although to a lesser extent (up to 1.6‐fold increases). Our study advances the notion that fish recruitment stability in estuaries is controlled by biocomplexity—life history diversity and spatiotemporal variation in the environment. However, intensified drought and marine heatwaves may increase the risk of multiple consecutive recruitment failures by synchronizing species dynamics and trajectories via Moran effects, potentially diminishing estuarine nursery function.

## Graphical abstract

Our analysis of long‐term population dynamics of juvenile fishes rearing in the San Francisco Estuary (California, USA) revealed that both biological and spatial ‘insurance’ mechanisms stabilized aggregate fish recruitment in the face of environmental variability over four decades. However, as climate change continues to intensify drought and marine heat waves, species dynamics may become synchronized, ultimately eroding stability of nursery function in the estuary.

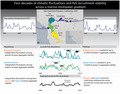

## 1 INTRODUCTION

Biological insurance and portfolio effects are key concepts in ecology, biodiversity conservation, and natural resource management (Loreau, 2000; MacArthur, 1955; Schindler et al., 2015). In a variable environment, biodiversity can stabilize whole communities when species with different biological traits fluctuate asynchronously in space, time, or both (Loreau et al., 2021). In fisheries, portfolio effects have been documented in marine and freshwater systems (Matsuzaki et al., 2019; Schindler et al., 2010; Thorson et al., 2018) and across biological levels of organization—from spatially structured populations (Carlson & Satterthwaite, 2011; Hilborn et al., 2003; Schindler et al., 2010) to communities (Anderson et al., 2017; Hammond et al., 2020). Mounting evidence suggests that portfolio effects can arise from biological structure (i.e., the combination of species with different biological traits) or spatial structure (i.e., heterogeneity in the environments they inhabit), which promote independent fluctuations and thus buffer the community from variability (Greene et al., 2010; Hilborn et al., 2003; Moore et al., 2014). However, the role of biocomplexity—complexity among species and environments—in stabilizing fish communities in estuaries, which are highly dynamic and complex transition zones that function as critical nursery areas for a vast array of fishes globally, has yet to be investigated.

Estuaries are highly productive ecosystems that function as nurseries by enhancing growth and survival of juvenile fishes across a wide variety of taxa, habitats, and environmental conditions (Beck et al., 2001; Nagelkerken et al., 2015). Fish distribution and abundance in estuaries may vary across spatial gradients (e.g., temperature, salinity) and temporal scales (e.g., tidal, diel, seasonal, multi‐annual; Lauchlan & Nagelkerken, 2020). Complex life cycles have evolved whereby species may reside in estuaries during specific life stages or move into them opportunistically when favorable conditions arise (Elliott et al., 2007). For example, common life history strategies include anadromy, whereby species are born in freshwater (e.g., rivers, streams), move downstream into estuaries (often to rear) and then the ocean (to grow), and return to freshwater to spawn; marine opportunism, whereby species opportunistically move into estuaries from the ocean; and estuarine dependence, whereby species reside in estuaries for a significant portion of their life cycles, especially during early life stages (Elliott et al., 2007). Due to the commercial, recreational, and ecological importance of fishes that use estuaries as nurseries globally, understanding the role of biodiversity in stabilizing aggregate fish recruitment in response to climatic variability is a critical conservation and management objective.

Under accelerating climate change, fishes that use estuaries are increasingly subject to cumulative impacts of multiple interacting stressors in fresh, brackish, and marine environments (Colombano et al., 2021; Lauchlan & Nagelkerken, 2020). Fish communities in estuaries tend to track climatic variability through space and time (Cloern et al., 2010; Feyrer et al., 2015; Pollack et al., 2011), suggesting that climate change may result in “winners” and “losers” (Somero, 2010). Multiple stressors may exceed species' physiological thresholds (Lauchlan & Nagelkerken, 2020), shift phenologies (Thaxton et al., 2020), amplify matches or mismatches with food resources (Asch, 2015; Chevillot et al., 2017), or exacerbate human impacts such as habitat loss (Moyle et al., 2013) or fishing pressure (Griffith et al., 2012). Extreme events may homogenize environmental gradients (e.g., strong storms that freshen the entire estuary or prolonged droughts that elevate salinity levels far upstream; Ghalambor et al., 2021), and in doing so, they may synchronize population dynamics via the Moran effect (i.e., regionally coordinated environmental fluctuations; Moran, 1953). Collectively, intensified climate change may favor some juvenile fish species based on their biological traits, while others may be at risk of multiple consecutive recruitment failures and associated increased extinction risk.

The potential ecological consequences of climate change on juvenile fishes in estuaries extend beyond the recruitment of individuals to adult populations. Juvenile fishes in estuaries are common prey resources for higher level consumers and thus often serve as critical linkages in food webs across the marine‐freshwater gradient (Deegan, 1993). Simulations of decreased fish production in marine food webs under climate change scenarios show severe negative effects on energy transfers to consumers and ecosystem production worldwide (du Pontavice et al., 2021). Recently, forage fish populations in the Gulf of Alaska severely declined in response to the Pacific marine heatwave of 2014–2016, which temporarily overwhelmed decadal‐scale climatic variability, and resulted in shifts in distribution, mass mortalities, and reproductive failures of seabirds, marine mammals, and groundfish (Arimitsu et al., 2021). These observed declines were apparent at the onset of the marine heatwave event, suggesting that forage fish declines may serve as early warning signs for food web and ecosystem instability (Arimitsu et al., 2021). Overall, there is a growing recognition that climate‐induced variability in juvenile fish abundance may have strong bottom‐up effects on food webs.

In this study, we examined spatiotemporal stability in juvenile fish abundance in the San Francisco Estuary, CA, USA. Our main goals were (1) to characterize patterns of abundance and environmental responses among species and life histories, and (2) to examine the role of biological and spatial insurance in buffering (i.e., reducing the temporal variability of aggregate fish abundance) at the community level. We first asked, how does juvenile (age 0) fish abundance vary over space, time, and in response to freshwater flow and sea surface temperature (SST)? Using multiple sources of monitoring data from 1980 to 2018, we modeled the abundance of juvenile fish species with diverse life history strategies and quantified the effects of freshwater flow and SST across five regions of the estuary. We expected abundance trends and environmental effects to vary widely based on species identity, estuarine use type, and thermal and salinity tolerances (Elliott et al., 2007; Feyrer et al., 2015; Teichert et al., 2017). Next, we asked, does biological or spatial structure buffer long‐term aggregate fish abundance from environmental variability? We hypothesized that buffering could arise from (1) biological insurance, whereby independent fluctuations among species and life histories reduce the temporal variability in aggregate juvenile fish abundance, or (2) spatial insurance, whereby independent fluctuations among spatial units reduce the temporal variability in aggregate juvenile fish abundance (Loreau et al., 2021). Finally, we explored the potential conservation implications of our findings under accelerating climate change.

## 2 METHODS

### 2.1 Study area

**FIGURE 1:**
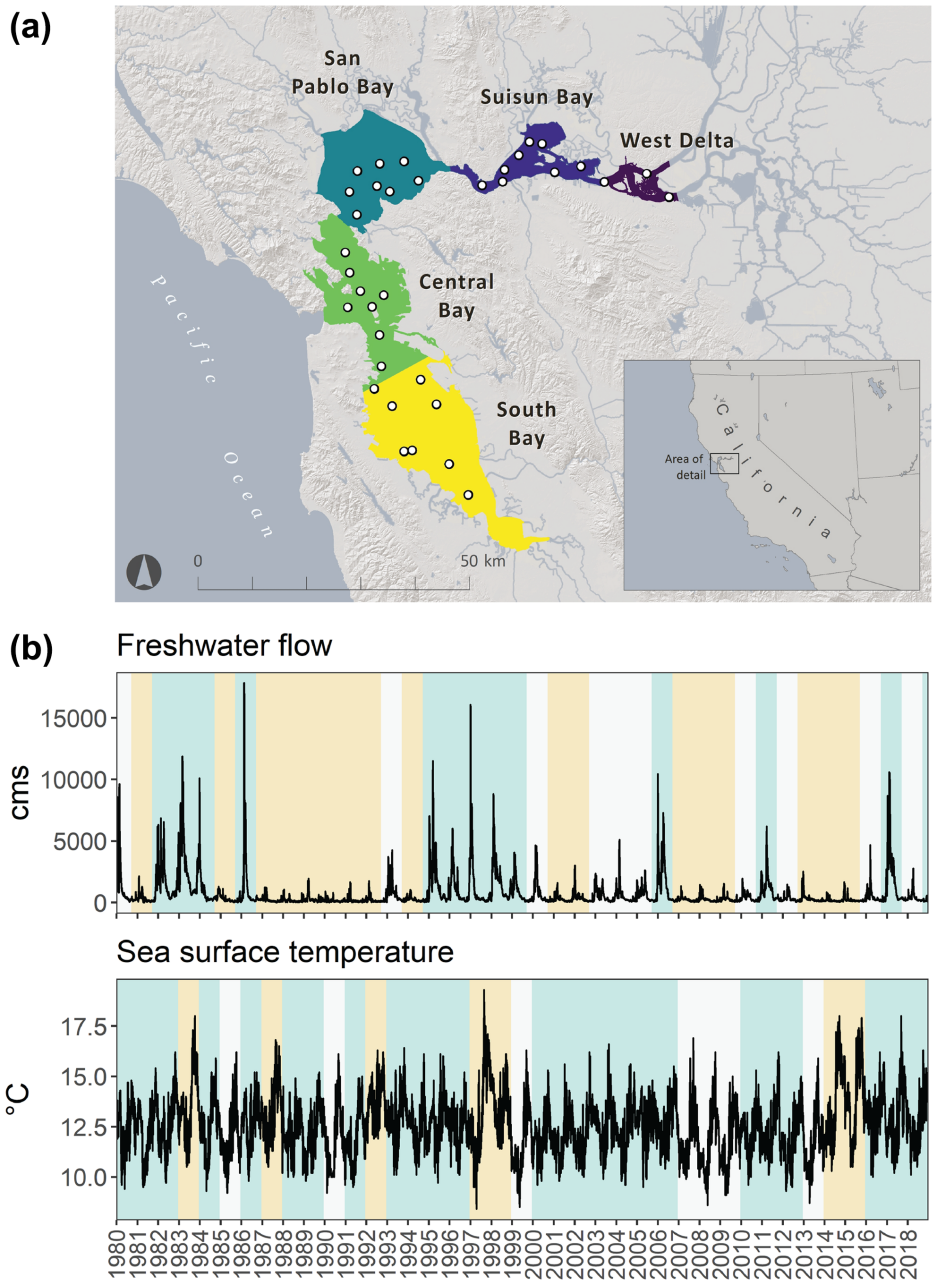
Study site and long‐term hydroclimatic context. (a) Map of the San Francisco Estuary, California, USA. Regions are shown as color‐coded polygons, and core fish sampling stations are shown as white circles. Along the longitudinal axis of the estuarine gradient, and depending on hydroclimatic conditions, salinity can range from fresh (e.g., West Delta) to salty (in the Central Bay, which is connected to the Pacific Ocean). Data source: CDFW (2020). Image credit: Amber Manfree. (b) Daily variation in freshwater flow (flow; m^3^ s^−1^) and sea surface temperature (SST; °C) from 1980 to 2018. Relative annual conditions (flow: Water year = October to September; SST: Calendar year = January to December) are shown as color‐coded vertical lines: White = average (within 1 *SD*); blue = below average (below −1 *SD*); yellow = above average (above 1 *SD*). Data sources: CDWR (2020) and UCSD (2020).

Survey data were obtained from the San Francisco Bay Study, a long‐term monitoring program that samples fishes along the longitudinal salinity gradient of the San Francisco Estuary (hereafter, ‘SF Estuary’), California, USA (Figure 1a; CDFW, 2020). The SF Estuary is a temperate estuary situated between the Pacific Ocean and the largest river system contained entirely within California, the Sacramento‐San Joaquin Delta (hereafter, “Delta”). The Delta drains roughly 40% of the state's freshwater and is highly regulated by dams and reservoirs that capture, store, and divert water for agricultural operations and municipalities (Cloern & Jassby, 2012). California's Mediterranean climate drives the magnitude and duration of seasonal precipitation in winter and spring, which influences the annual freshwater flow entering the SF Estuary, its interaction with tidal waters from the Pacific Ocean, and in turn, the location and extent of the salinity gradient (Gross et al., 2009; Kimmerer et al., 2013). Generally, the upstream regions are fresh (0 Practical Salinity Units, PSU); the regions in the center of the gradient are brackish (0–15 PSU); and the downstream regions are mesohaline (5–18 PSU) or polyhaline (18–30 PSU). However, during extreme wet years, freshwater can extend as far west as the Golden Gate strait at the entrance of the Pacific Ocean; alternatively, during extreme dry years, low salinity water (3–5 PSU) can encroach as far inland as the interior Delta, prompting emergency management actions to protect freshwater supplies (Sommer, 2020). The highly variable salinity gradient is the subject of extensive management efforts and provides an opportunity to study ecological responses of estuarine transition zones to climate forcing over ocean and river systems (see Cloern & Jassby, 2012; Cloern et al., 2010; Feyrer et al., 2015; Raimonet & Cloern, 2017).

According to the Intergovernmental Panel on Climate Change's Sixth Assessment Report, climate change is currently altering atmospheric and oceanic processes (e.g., circulation patterns, temperature, salinity, evapotranspiration, precipitation, flooding, aridity, drought) on global and regional scales (IPCC, 2021). In the SF Estuary, the combination of multiple consecutive dry and warm winters with reduced precipitation (i.e., rainfall, snow) and less frequent reservoir releases to maximize water storage have become more frequent, resulting in extreme, prolonged droughts in the SF Estuary (Knowles & Cronkite‐Ratcliff, 2018; Pierce et al., 2018; Reis et al., 2019). Rising sea levels may elevate baseline salinity levels throughout the year and particularly during summer, while drought conditions may cause extreme salinity intrusion due to extended periods of low flows and tidal mixing farther upstream (Ghalambor et al., 2021). These extreme dry years are expected to be interspersed with extreme wet years featuring intensified atmospheric rivers that bring most of the precipitation in the form of rainfall rather than snow (Dettinger et al., 2016).

Ocean climate patterns such as the Pacific Decadal Oscillation (PDO), the North Pacific Gyre Oscillation (NPGO), and the El Niño Southern Oscillation are characterized by alternating phases of ocean currents and SSTs (Di Lorenzo et al., 2008; Mantua & Hare, 2002). SSTs in the Gulf of Farallones fluctuate synchronously with water temperatures in the lower SF Estuary (Raimonet & Cloern, 2017), and strong upwelling events with cooler SSTs are linked to higher primary and secondary production in the lower SF Estuary (Cloern & Jassby, 2012). Recent SST trends show directional increases and more frequent and severe anomalies (i.e., “marine heatwaves”), which are predicted to further intensify over the next century as climate change progresses (Fox‐Kemper et al., 2021). In combination, freshwater flows (Flow) and SSTs are key indicators of hydrologic and oceanic variability, respectively, that bookend estuarine transition zones—both in the SF Estuary (Cloern & Jassby, 2012; Feyrer et al., 2015) and in estuaries worldwide (Colombano et al., 2021; Lauchlan & Nagelkerken, 2020).

### 2.2 Environmental sensor data and metrics

To describe environmental variables that characterize freshwater versus marine influence in estuaries, we used publicly accessible data from multiple long‐term environmental monitoring programs. Freshwater flow was based on “Net Delta Outflow,” a metric that is derived from a widely used hydrologic model that uses flow sensor data to estimate mean daily flows exiting the Delta and entering the San Francisco Bay (CDWR, 2020). Daily SST measurements were acquired from the Scripps Institute of Oceanography's Shore Stations Program site located at the Farallon Islands (26 miles west of the Golden Gate strait in the Pacific Ocean; UCSD, 2020). Both Flow and SST time series (Figure 1b) have been used extensively in previous ecological analyses in the SF Estuary (Cloern & Jassby, 2012; Feyrer et al., 2015; Goertler et al., 2021; Sydeman et al., 2018).

For inclusion in multivariate autoregressive state‐space (MARSS) models (see Sections 2.4, 2.6), we prescreened the covariates (Figure S1). We calculated mean annual flow and SST from April to October 1980 to 2018 for consistency with the fish monitoring data (see Section 2.3). We assessed the correlation between the April to October window (when fish sampling is most consistent) and the January to June window (when the bulk of California's precipitation occurs) to confirm that the April–October window sufficiently captured annual trends (Pearson's *r* = .905). For missing SST data, we applied a seasonal autoregressive integrated moving average model with a Kalman filter to interpolate missing or flagged values before summarizing the annual means (Figure S2; Comte et al., 2021). We checked for multicollinearity between flow and SST by examining variance inflation factors (criteria <2; Fox et al., 2013), and assessing correlation between the two. Because these metrics were only weakly correlated (Pearson's *r* = .298), we deemed both “Flow” and “SST” metrics appropriate for inclusion in the models.

### 2.3 Fish sampling and life history classification

The San Francisco Bay Study is a long‐term monitoring program initiated in 1979 that samples 35 core fixed stations on a monthly basis across five regions spanning the salinity gradient: the West Delta, Suisun Bay, San Pablo Bay, Central Bay, and South Bay (Figure 1a). Fish sampling is conducted with an otter trawl to target benthic species and a midwater trawl to target pelagic fishes in open water habitats of the estuary. Captured species are identified, measured, and enumerated, and sampling effort (otter trawl: area‐swept [m^2^]; midwater trawl: volume [m^3^]) is recorded to standardize catch metrics (i.e., catch per unit effort or “CPUE”; CDFW, 2020). We included 39 years (1980–2018) of age‐0 fish data collected from April to October, which encompasses the most consistently sampled months by both gear types, and adequately captures peak abundances for common fish species during the age‐0 life stage (Figure S3; Feyrer et al., 2015). Life history classification (anadromous, marine opportunist, or estuarine dependent) was based on species‐level life cycles and patterns of estuarine use and migration in the SF Estuary or other California coastal waters (Table 1; Table S1; Allen & Horn, 2006; Elliott et al., 2007; Moyle, 2002).

**TABLE 1:** Summary of 18 fish species, life history type, gear type, and capture location. Species are listed by common name, Latin name, origin (native vs. nonnative), and dominant life history based on estuarine use (Allen & Horn, 2006; Elliott et al., 2007; Moyle, 2002). Gear type and capture location reflects the California Department of Fish and Wildlife's San Francisco Bay study monitoring program data collected from 1980 to 2018. Gear type codes: MWT, midwater trawl; OT, otter trawl. Region codes: C, Central Bay; S, South Bay; SP, San Pablo Bay; SU, Suisun Bay; W, West Delta. See Table S1 for species' associated salinity and temperature ranges

Code	Species	Latin name	N/NN	Life history: estuarine use type	SF Bay study: gear type(s)	SF Bay study: region(s)
LONSME	Longfin Smelt	*Spirinchus thaleichthys*	N	Anadromous	MWT, OT	C, SP, SU, W
STRBAS	Striped Bass	*Morone saxatilis*	NN	Anadromous	MWT, OT	SP, SU, W
AMESHA	American Shad	*Alosa sapidissima*	NN	Anadromous	MWT	SP, SU, W
CALTON	California Tonguefish	*Symphurus atricauda*	N	Marine opportunist	OT	S, C
BROROC	Brown Rockfish	*Sebastes auriculatus*	N	Marine opportunist	OT	S, C
SPESAN	Speckled Sanddab	*Citharichthys stigmaeus*	N	Marine opportunist	OT	S, C, SP
ENGSOL	English Sole	*Parophrys vetulus*	N	Marine opportunist	OT	S, C, SP
NORANC	Northern Anchovy	*Engraulis mordax*	N	Marine opportunist	MWT	S, C, SP, SU
WHICRO	White Croaker	*Genyonemus lineatus*	N	Estuarine dependent	OT	S, C, SP
YELGOB	Yellowfin Goby	*Acanthogobius flavimanus*	NN	Estuarine dependent	OT	SP, SU, W
BAYGOB	Bay Goby	*Lepidogobius lepidus*	N	Estuarine dependent	OT	S, C, SP
SHIPER	Shiner Surfperch	*Cymatogaster aggregata*	N	Estuarine dependent	MWT, OT	S, C, SP
STAFLO	Starry Flounder	*Platichthys stellatus*	N	Estuarine dependent	OT	SP, SU, W
PACHER	Pacific Herring	*Clupea pallasii*	N	Estuarine dependent	MWT	S, C, SP, SU
PLAMID	Plainfin Midshipman	*Porichthys notatus*	N	Estuarine dependent	OT	S, C, SP, SU
JACKSM	Jacksmelt	*Atherinopsis californiensis*	N	Estuarine dependent	MWT	S, C, SP
PACSSC	Pacific Staghorn Sculpin	*Leptocottus armatus*	N	Estuarine dependent	OT	S, C, SP, SU, W
WALSUR	Walleye Surfperch	*Hyperprosopon argenteum*	N	Estuarine dependent	MWT	C

### 2.4 Criteria for inclusion of fish data in MARSS models

We analyzed the fish abundance time series using MARSS models (Holmes et al., 2012) and examined the effects of Flow and SST on age‐0 fish abundance, while accounting for multiple gear types. To meet data density requirements for model convergence, we filtered the fish data set to retain “frequent” age‐0 species, retaining those occurring in 50% of the time steps at a given station, with a minimum of one station and one gear type required to represent a region. The 18 frequent age‐0 species (Table 1) of 22 total age‐0 species were modeled using station‐ and gear‐level observation data to estimate “states” (i.e., observation error free abundance fluctuations based on CPUE) in each region.

### 2.5 MARSS model specifications

The MARSS approach is a more sophisticated, state‐space version of the multivariate autoregressive (MAR) approach. MAR is commonly used for ecological time series analysis because it can estimate the effects of environmental variables, biotic interactions (Hampton et al., 2013), and spatial structure (Ward et al., 2010). Expanding on MAR, MARSS has equations for a state process (Equation 1) to estimate the “true” fluctuations, and an observation process (Equation 2) to account for measurement error while also accommodating missing data (Holmes et al., 2012). Modeling observation error is particularly important for long‐term data sets, which tend to have noisy observations owing to variation in detectability or sampling methodology and, if ignored, could lead to incorrect statistical inference (Knape & de Valpine, 2012). In the matrix form, MARSS model specifications for each species were specified as follows:
(1)

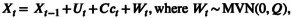

(2) $$Y_{t}=ZX_{t}+V_{t},\text{where}\;V_{t}∼\mathrm{MVN}\left(0,R\right),$$

where station‐ and gear‐specific age‐0 CPUE are the observations that enter the model in Equation 2 as *Y*_*t*_, where *t* is time. Gear types within a region (i.e., otter trawl, midwater trawl) inform the same state via a custom‐made *Z* matrix, which connects observations to states and provides an observation error variance value per region and gear type (in *R*, the observation error variance–covariance matrix). In Equation (1), the environmental covariates (i.e., Flow, SST) enter the model as *c*_*t*_; *C* is a matrix that captures the covariate effects, and *U* the long‐term average population growth. In turn, *W*_*t*_ is a matrix of the process error that captures deviations due to demographic or (unmeasured) environmental stochasticity, with process errors at time *t* being multivariate normal (MVN) with mean 0 and covariance matrix *Q*. We estimated all possible parameters in this *Q* matrix (using the “unconstrained” setting), as residual spatial covariance (SpCov) could be expected among regions. Overall, this model structure yields regional estimates of the *X*_*t*_ states and quantifies the unique effects of the environment via the *C* matrix, while controlling for other sources of stochasticity—either real (in the *Q* matrix) or potential, like measurement error in the data (via the *R* matrix).

To explore the diversity of responses of age‐0 fishes to key environmental drivers, we initially constructed a series of MARSS models for each species with the following covariates: Flow only; SST only; both Flow and SST; and no covariates. We also explored the influence of water clarity (measured with a Secchi disk, hereafter “Secchi”) on detectability by specifying it as a covariate in the observation model. However, its inclusion did not fundamentally change our inferences about Flow and SST effects on age‐0 fish abundance (see Table S2 for all model comparisons, and Figure S4). Here, we focus on the two main models that bookend the model comparison: (1) a “null” model with no environmental covariates, and (2) the “full” model with the additive terms of Flow and SST. We interpreted consistent significant effects of Flow or SST on abundance within and among species and life histories as the potential for synchronization by the environment, or Moran effects.

### 2.6 Model fitting and diagnostics

For each species model, age‐0 CPUE data were natural log(*x* + 1) transformed, and environmental covariates were *z*‐scored. Using the r “*MARSS*” package (Holmes et al., 2021), models were fitted using maximum likelihood estimation maximization algorithm (the Kalman filter) run for up to 5000 iterations each time. All model coefficients were evaluated based on bootstrapped 95% confidence intervals (CIs), where *C*, *Q*, and *R* intervals overlapping with zero were interpreted as non‐significant. Model comparisons of the null versus full models were assessed via corrected Akaike information criteria (AICc) using the Program r ‘MuMIn’ package (Bartoń, 2020).

### 2.7 Evaluating portfolio effects via biological and spatial structure

To compare the roles of biological and spatial insurance in reducing the temporal variability of aggregate fish abundance, we characterized long‐term abundance trends and CVs (or the standard deviation divided by the mean) on the states obtained from the MARSS models. First, we used the non‐parametric Theil–Sen (TS) estimator, a method that is robust to outliers, to determine whether species abundance trends in each region and across the estuary were increasing, decreasing, or dynamically stable. We based the calculations on the states generated by the MARSS models instead of the raw CPUE because they account for both process and observation error. Second, we calculated CVs for each biological and spatial group: individual species in regions; individual species across the estuary; aggregate life histories in regions; aggregate life histories across the estuary; the aggregate community in regions; the aggregate community across the estuary (see Table 2 for definitions). Finally, we calculated scaling factors as CV ratios to represent the change in mean CV with each change in biological or spatial scale. For example, the ratio between the mean CV for species across the estuary and the mean CV for life histories across the estuary represents an increase in biological scale. We interpreted reductions in CVs as evidence of buffering and a portfolio effect (Schindler et al., 2010).

**TABLE 2:** Terminology and definitions for each biological and spatial group considered in the portfolio effect analysis. Species in regions are the most disaggregated of the groups (*n* = 53) whereas the whole community across the estuary is the most aggregated of the groups (*n* = 1)

Term	Biological group	Spatial group	Definition	# groups
Species (region)	Species	Region	Total number of species and region combinations	53
Species (estuary)	Species	Estuary‐wide	Total number of species across the estuary	18
Life history (region)	Life history	Region	Total number of life history and region combinations	13
Life history (estuary)	Life history	Estuary‐wide	Total number of life histories across the estuary	3
Community (region)	Community	Region	Total number of community and region combinations	5
Community (estuary)	Community	Estuary‐wide	Total number of communities across the estuary	1

## 3 RESULTS

### 3.1 Climate‐driven hydrologic and oceanic variability influencing the estuary

Trends over the 39‐year data sets revealed substantial interannual variation in both Flow and SST (Figure 1b). As expected of a system influenced by California's Mediterranean hydroclimate, the Flow time series encompassed multiple consecutive dry years (e.g., 1987–1992, 2012–2016); however, earlier in the time series, these droughts were interspersed with multiple consecutive wet years (e.g., 1982–1984; 1995–1999), and were later punctuated by singular, extreme wet years (e.g., 2006, 2011, 2017) (Herbold et al., 2022). Typical of Eastern Pacific Ocean climate oscillations, the SST time series encompassed cool and warm phases. Notably, the time series captured a prominent phase shift to a highly productive PDO−/NPGO+ regime in the late 1990s (Cloern & Jassby, 2012) and anomalous marine heatwaves (e.g., the Pacific marine heatwave of 2014–2016; Cavole et al., 2016). Importantly, the time series captured all different possible combinations of Flow (wet/dry) and SST (warm/cool) phases, representing different types of hydrologic and oceanic variability observed over the past four decades.

### 3.2 Age‐0 species and life history level trends in abundance over space and time

**FIGURE 2:**
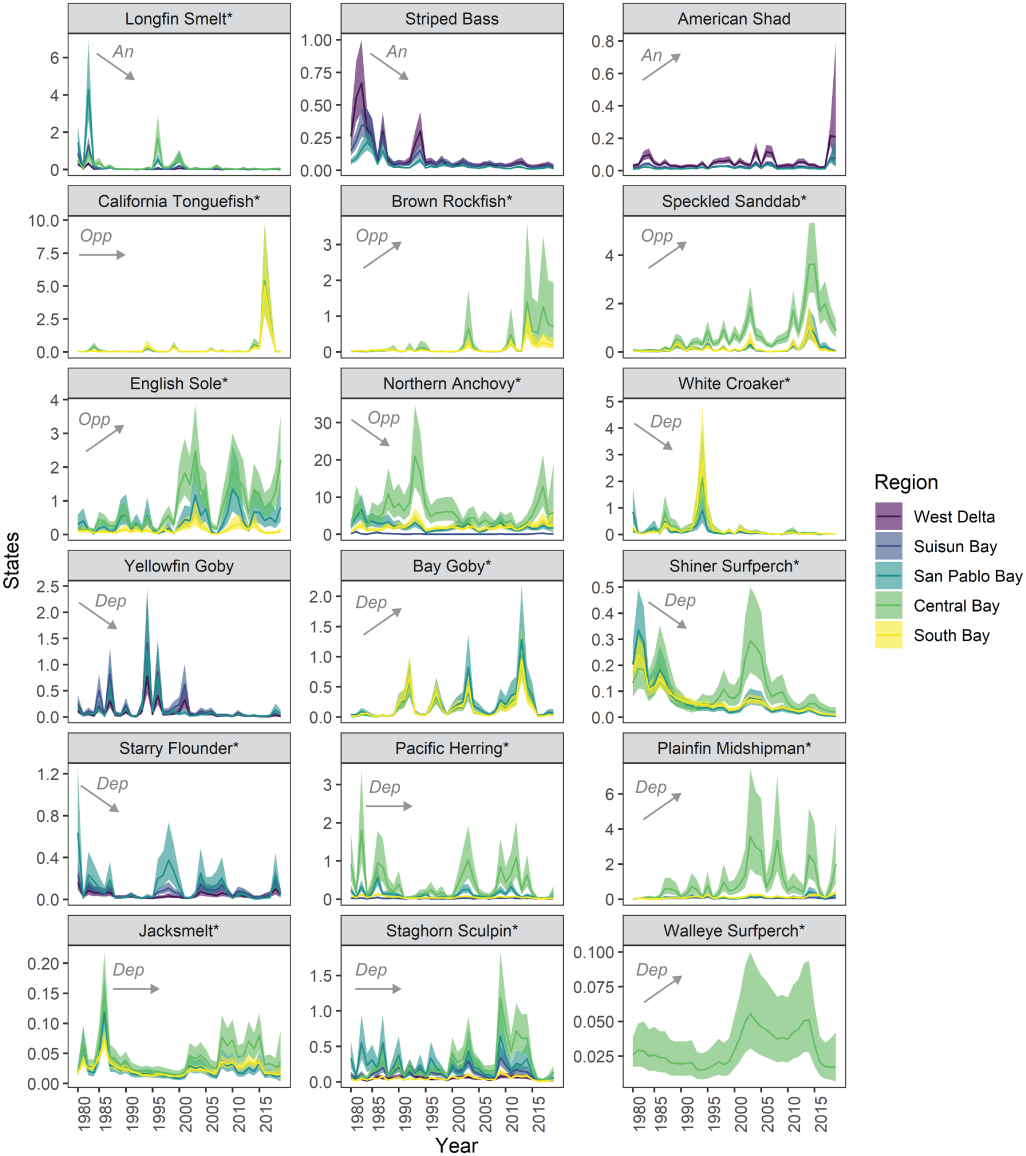
Long‐term trends in abundance of age‐0 species in each region. States were estimated with catch‐per‐unit‐effort data from 1980 to 2018 using multivariate autoregressive state‐space models. Regions are color‐coded. Estuarine use types are abbreviated as: An, anadromous; dep, estuarine dependent; Opp, marine opportunist. Native species are marked with an asterisk. Statistically significant (*p* < .05) Theil–Sen (TS) robust trends across the estuary are shown as increasing or decreasing with upward and downward trending arrows, respectively, and non‐significant trends are shown as flat arrows (see Table S3 for region‐specific TS estimates and Section 2 for details).

Estimated states from the MARSS models yielded a variety of trends among the frequent age‐0 species, life histories, and regions (Figure 2; Tables S2 and S3). Broad‐scale spatial patterns emerged for species according to their life history type across the estuarine gradient. Anadromous species, representing 3 of 18 total species and ~5% total catch, were common in the upper estuary (e.g., West Delta, Suisun Bay). Marine opportunist species, representing 5 of 18 species and ~72% estimated catch, were common in the lower estuary (e.g., South Bay, Central Bay). Estuarine dependent species, representing 10 of 18 of species and ~23% estimated catch, were common throughout the estuary (i.e., often found in 4 out of 5 regions).

At the estuary scale, the abundances of anadromous Longfin Smelt (*Spirinchus thaleichthys*) and Striped Bass (*Morone saxatilis*) significantly declined over time (TS: −0.12 and −0.15) but increased slightly for American Shad (*Alosa sapidissima*; TS: 0.02). Northern Anchovy (*Engraulis mordax*) significantly declined on average, except in South Bay where it increased (TS: −3.33 and 2.00). In contrast, the estuarine‐dependent species, Pacific Herring (*Clupea pallasii*), Jacksmelt (*Atherinopsis californiensis*), and Pacific Staghorn Sculpin (*Leptocottus armatus*), exhibited “boom and bust” cycles, with no consistent abundance trends. In turn, marine opportunists such as Brown Rockfish (*Sebastes auriculatus*), Speckled Sanddab (*Citharichthys stigmaeus*), and English Sole (*Parophrys vetulus*) tended to increase in abundance over time (TS: 0.18, 1.11, and 1.36). Overall, we observed a diversity of spatial distributions (narrow vs. widespread) and abundance trends (increasing, decreasing, and dynamically stable) across species and regions, which were largely explained by life‐history strategies.

### 3.3 Differential responses to freshwater flow and SST

**FIGURE 3:**
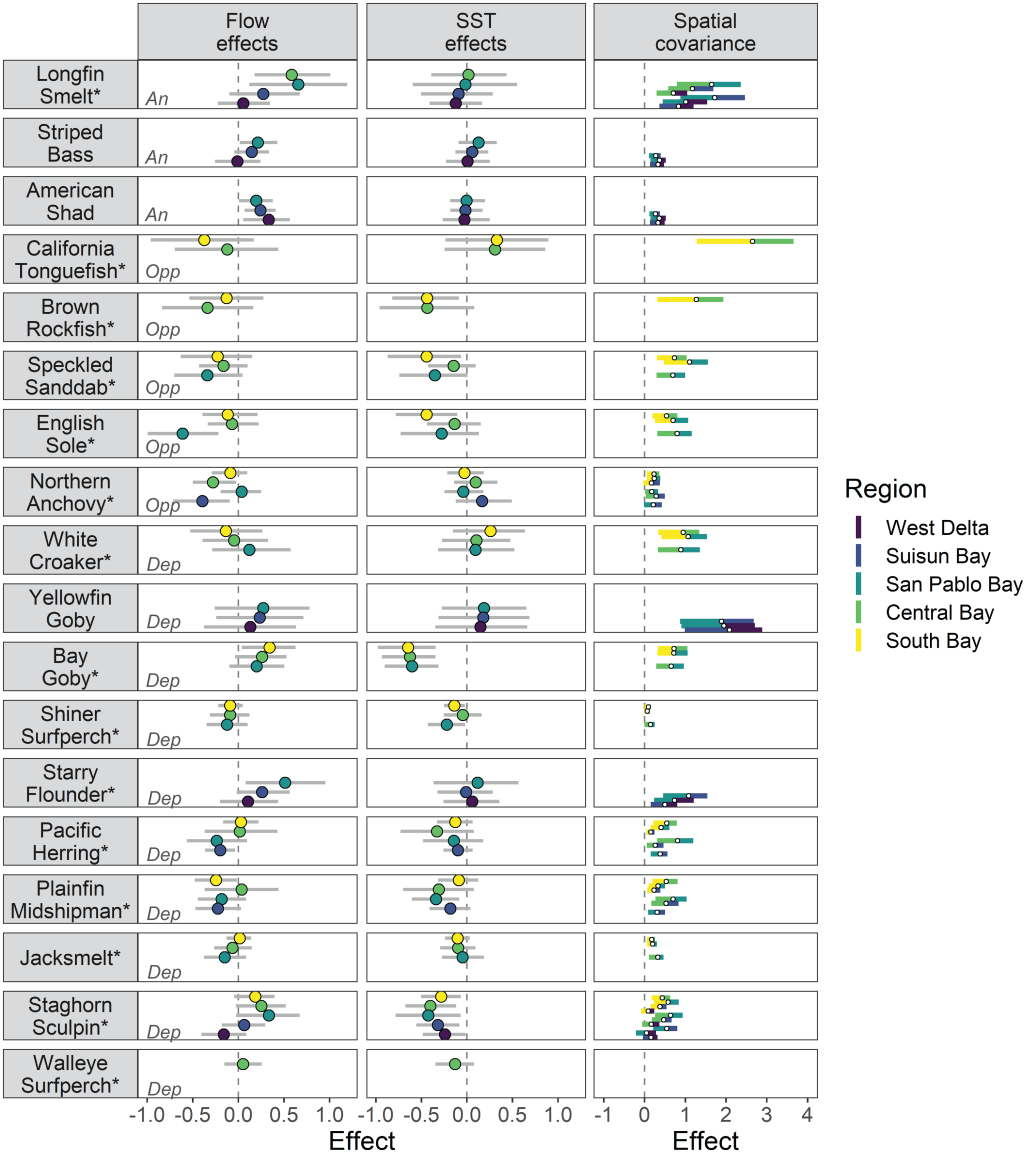
Effects of interannual variation in freshwater flow and sea surface temperature, and residual spatial covariance, on age‐0 fish abundance. Coefficients for each age‐0 species and region were estimated using multivariate autoregressive state space models (see Section 2 for details). Bootstrapped confidence intervals (95%) excluding zero can be interpreted as statistically significant. Estuarine use types are abbreviated as: An, anadromous; Dep, estuarine dependent; Opp, marine opportunist. Native species are marked with an asterisk.

Species models with covariate effects were in 83% of cases top‐ranked in model comparisons (Table 3). Species had different combinations of positive, negative, and no responses to Flow and SST, and several patterns emerged for life history types (Figure 3). Anadromous species showed positive relationships to Flow but no relationship to SST; however, Flow effects varied geographically. Longfin Smelt and Striped Bass showed increasingly stronger positive relationships to Flow further downstream in the estuarine gradient (e.g., stronger in San Pablo Bay), whereas American Shad showed the opposite relationship (e.g., stronger in the West Delta). Marine opportunist species showed a range of Flow and SST effects. For example, Brown Rockfish, Speckled Sanddab, and English Sole in the South Bay responded positively to cooler SSTs, and English Sole and Northern Anchovy in the upper estuary responded positively to lower flows (i.e., drought). Estuarine dependent species showed the greatest diversity in responses to Flow and SST: half of the species showed no response to one or more covariates while the other half showed responses to all different combinations. Notably, the estuarine dependent species that showed significant SST relationships were all associated with cooler conditions (e.g., Pacific Staghorn Sculpin, Bay Goby (*Lepidogobius lepidus*), Plainfin Midshipman (*Porichthys notatus*), Shiner Surfperch (*Cymatogaster aggregata*), and Jacksmelt). In contrast, several species showed no covariate effects (e.g., White Croaker (*Genyonemus lineatus*), Walleye Surfperch (*Hyperprosopon argenteum*), Yellowfin Goby (*Acanthogobius flavimanus*)). All species showed significant process error covariance, representing spatial synchrony that was not accounted for by Flow or SST (Figure 3; Figure S5). Altogether, these results confirmed age‐0 species responsiveness to both environmental drivers, diversity in the direction and magnitude of responses, and the potential for these two environmental drivers to synchronize trends across large spatial scales via the Moran effect.

**TABLE 3:** Comparison of multivariate autoregressive state space models with and without environmental covariates. We compare null models (without covariates) to full models (with flow and sea surface temperature as covariates, and residual spatial covariance from the process error covariance matrix) via an information‐theoretic approach using the corrected Akaike information criterion (AICc). See Table S2 for complete model comparisons and parameter estimates and Section 2 for details

Species	Null model (AICc)	Full model (AICc)
Longfin Smelt	3851	3734
Striped Bass	3138	3077
American Shad	1276	1195
California Tonguefish	1022	957
Brown Rockfish	302	290
Speckled Sanddab	2833	2765
English Sole	3543	3479
Northern Anchovy	3801	3791
White Croaker	2225	2186
Yellowfin Goby	2145	2028
Bay Goby	3134	3030
Shiner Surfperch	2070	2055
Starry Flounder	1126	1075
Pacific Herring	3447	3378
Plainfin Midshipman	3320	3288
Jacksmelt	2034	1987
Pacific Staghorn Sculpin	4298	4192
Walleye Surfperch	111	115

### 3.4 Patterns of juvenile recruitment stability

**FIGURE 4:**
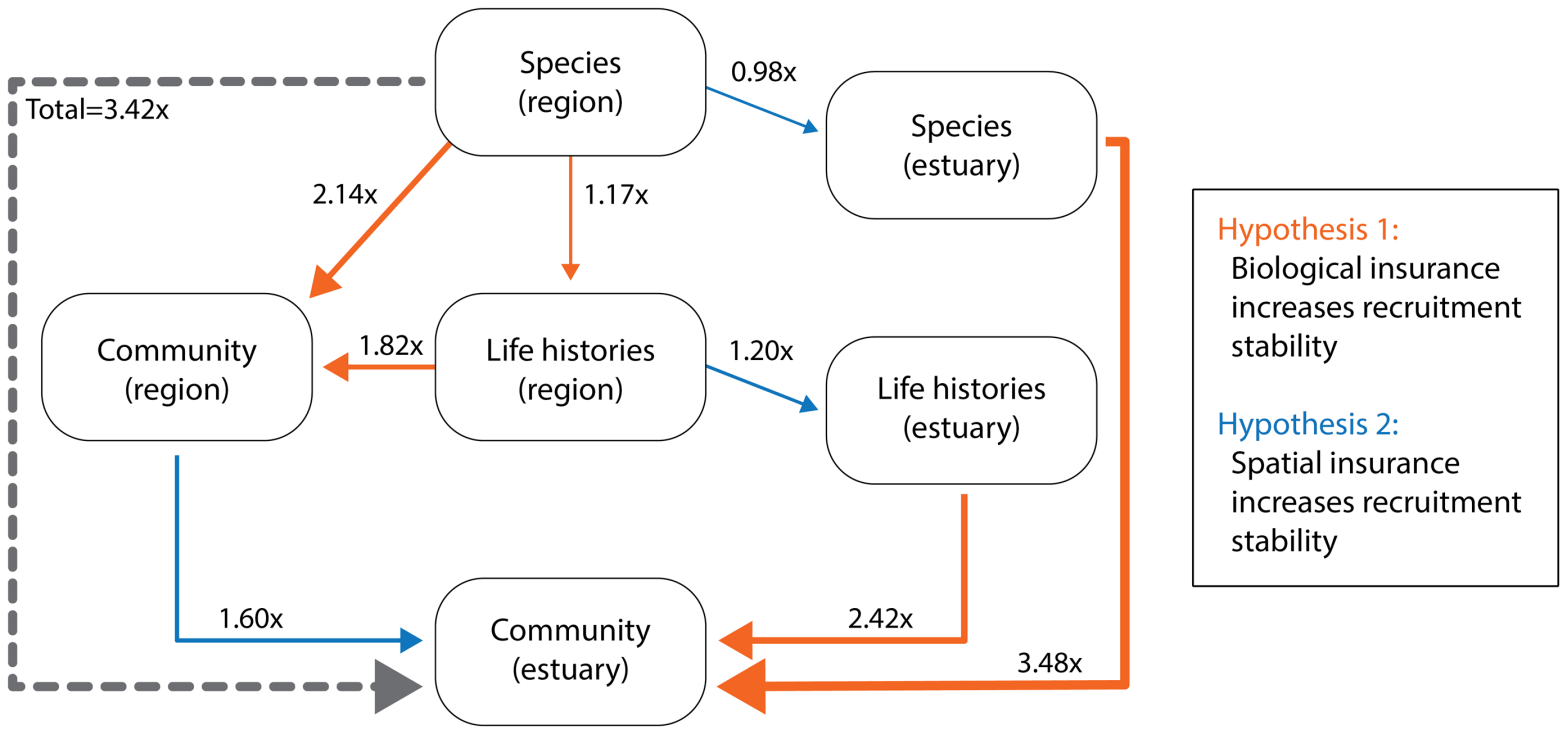
Summary of portfolio effects. We hypothesized two ways in which aggregate stability of the juvenile fish community could emerge: (1) “biological insurance” or independent fluctuations among species and life histories or (2) “spatial insurance” or independent fluctuations among regions of the estuary. Arrow colors represent the different pathways and widths represent the strength of the stability scaling factors. Stability scaling factors represent changes in the mean coefficient of variation (CV) from one group to the next. For example, scaling from Species (region) to Life histories (region) tests hypothesis 1 about biological insurance. Alternatively, scaling from Species (region) to Species (estuary) tests hypothesis 2 about spatial insurance. The CVs were calculated based on the states obtained from multivariate autoregressive state‐space models. See Section 2 for details.

**FIGURE 5:**
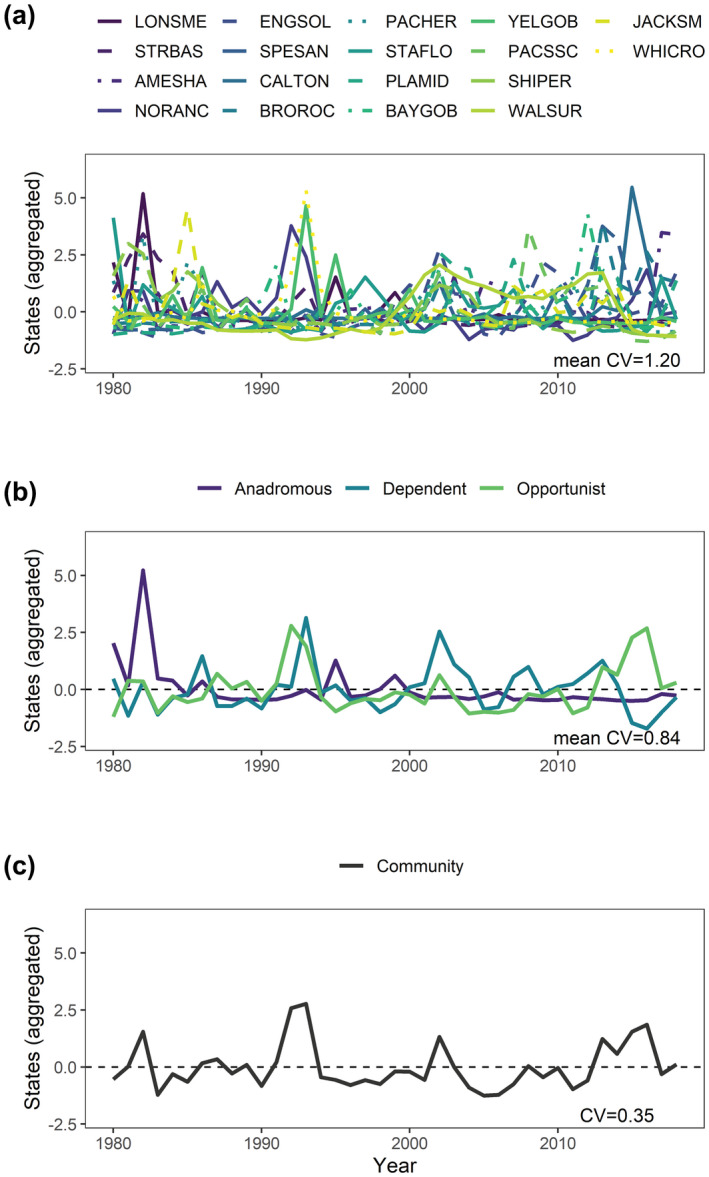
Biological insurance strongly buffered temporal variability in the juvenile fish community. Lines represent temporal variability of aggregated states for (a) species across the estuary, (b) life histories across the estuary, and (c) the whole fish community across the estuary. In all cases, to allow for meaningful comparison across levels of aggregation, states were *z*‐scored (*M* = 0; *SD* = 1). A reduction in coefficients of variation (CVs) from a to b to c is interpreted as evidence of strong portfolio effects arising from biological insurance. See Figure 4 for a diagram on the scaling of these mean CV values, Table 1 for fish codes and Section 2 for details.

Overall, the estuary‐wide juvenile fish community had the lowest CV and was 3.43 times more stable than species in regions on average (Figure 4). Scaling factors showed that both hypothesized factors contributed to this phenomenon: variation among biological and spatial groups. However, biological factors had a stronger buffering effect overall. While spatial scaling from biological groups in regions to biological groups across the estuary maintained or moderately increased stability (0.98–1.60), biological scaling from species and life histories across the estuary to the whole community across the estuary resulted in a 3.48‐fold and 2.42‐fold increase in stability, respectively (see CV decreases in Figure 5). While anadromous species were highly variable overall (CV: 1.59), mean abundance declined over time, resulting in increased stability in the latter half of the time series. In contrast, marine opportunist and estuarine dependent species were more stable overall (CV: 0.45 and 0.47, respectively), and showed variable levels of asynchrony with each other, with high negative covariance in recent years (Figure 5b). These results suggest that independent fluctuations among species and life histories provided strong biological insurance to the aggregate juvenile fish community.

## 4 DISCUSSION

In this study, we investigated broad‐scale spatial and temporal patterns of juvenile fish abundance and environmental data collected over four decades in the SF Estuary using spatially structured MARSS models. We addressed two main questions: (1) How does juvenile fish abundance vary over space, time, and in response to environmental variability? (2) Does biological and/or spatial insurance buffer long‐term aggregate juvenile fish abundance from environmental variability? Our models for 18 age‐0 fishes accounted for process error variance and covariance, observation error, and two environmental covariates (freshwater flow and SST). We found that species exhibited diverse abundance patterns in space and time, and largely responded to interannual fluctuations in flow and/or SST. Multiple mechanisms providing portfolio effects emerged, whereby diversity among species and among life histories were the strongest stabilizers (2.42 and 3.48‐fold increases in aggregate stability, respectively). Regional asynchrony was also a stabilizer, although to a lesser extent (up to 1.60‐fold increases in aggregate stability). These findings highlight the role of biocomplexity in buffering the juvenile fish community from environmental variability across the marine‐freshwater gradient. However, they also provide insight on the potential for climate extremes (e.g., droughts, marine heatwaves) to synchronize species dynamics and trajectories via Moran effects (Arimitsu et al., 2021; Koenig, 2002; Moran, 1953), which could lead to multiple consecutive recruitment failures and weakened portfolio effects in the estuary.

### 4.1 Abundance fluctuations over space, time, and in response to freshwater flow and SST

Abundances of age‐0 fishes (or “young of the year”) fluctuated in space and time, often in response to flow and SST. Estuarine dependent species had the greatest diversity of responses and were relatively common across the estuarine gradient. As is common in estuaries, anadromous species comprised the smallest percentage of total species (Franco et al., 2008). The observed declines in Longfin Smelt and Striped Bass, which frequently feed on zooplankton and rely on fresher conditions for spawning and rearing, have been thoroughly documented by several long‐term monitoring programs (Feyrer et al., 2007; Hobbs et al., 2006; MacNally et al., 2010; Nobriga & Rosenfield, 2016; Thomson et al., 2010). Freshwater flow effects were similar for both species, whereby abundances increased in response to freshening of the lower estuary (e.g., San Pablo Bay), which expands the availability of low salinity rearing habitat along the migration route to the Pacific Ocean (Grimaldo et al., 2020; Hobbs et al., 2006; Kimmerer et al., 2013). In contrast, American Shad remained in low abundance until recent years and responded positively to higher flows in the upper estuary (e.g., during 2017, the highest water year on record), mirroring observations in an adjacent brackish wetland (O'Rear et al., 2021). Overall, the abundances of all anadromous species and some estuarine dependent species (e.g., Starry Flounder [*Platichthys stellatus*], Bay Goby, Pacific Staghorn Sculpin) were synchronized by interannual fluctuations in freshwater flows (i.e., higher abundances in wet years). For the flow‐dependent anadromous species, this represents a largely unexplored, yet consequential form of the Moran effect (Koenig, 2002; Moran, 1953; Ranta et al., 1997), as it likely influences subsequent dynamics of these populations after they migrate to the ocean.

The observed increases in marine opportunist species over the latter half of the time series occurred in the lower estuary (e.g., South and Central Bay), which is connected to the Pacific Ocean and exhibits synchronous temperature fluctuations with coastal waters (Raimonet & Cloern, 2017). Early life stages of marine opportunist species (e.g., English Sole, Speckled Sanddab, Brown Rockfish) likely enter the estuary during cooler years when ocean productivity is higher due to coastal upwelling in the PDO−/NPGO+ phase (Cloern & Jassby, 2012). This may explain the significant SST effects in South Bay, a highly productive marine lagoon that can rapidly change in temperature due to its shallow embayments and sensitivity to atmospheric and oceanic forcing (Cloern & Jassby, 2012). In contrast, California Tonguefish (*Symphurus atricauda*) and Northern Anchovy were highly abundant during the concurrent record drought and marine heatwave of 2015. Pacific Herring, an estuarine dependent species, exhibited boom and bust cycles, typical of small pelagic fishes with short life cycles (McClatchie et al., 2017; Sydeman et al., 2018). The marine opportunist English Sole and estuarine dependent Pacific Herring and Plainfin Midshipman likely move upstream in dry years when low flows, tidal mixing, and salinity intrusion expand the upper extent of their range (e.g., into Suisun Bay). These findings align with previous research demonstrating that in this river‐dominated estuary, freshwater flow effects on fishes propagate further downstream (i.e., to marine fishes) than ocean‐derived metrics propagate upstream (i.e., to freshwater fishes; Feyrer et al., 2015), due to a decoupling of ocean‐estuary dynamics further inland (Raimonet & Cloern, 2017). Furthermore, they indicate that SST may also synchronize dynamics and trajectories of several cool‐tolerant marine opportunist and estuarine dependent species that rear in the lower estuary.

### 4.2 Factors buffering temporal variability of the aggregate juvenile fish community

Overall, the diversity of abundance responses to environmental fluctuations among species, life histories, and to some extent regions, helped stabilize the whole juvenile fish community over the 39‐year time series. Despite multiple shifts in atmospheric and oceanic climate patterns (i.e., wet/dry and cool/warm conditions), the diversity of life histories among the 18 species reduced temporal variability of aggregate fish abundance across the estuary. It is important to note, however, that there is a “mean‐stability tradeoff,” whereby increased stability may also occur when the mean abundance of a species is reduced due to population decline (Loreau et al., 2021). In this scenario, dampened variability in population fluctuations may result from a combination of multiple stressors or vulnerability to Moran effects. This pattern emerged for anadromous species, particularly for Longfin Smelt and Striped Bass, which are indicator species for the “pelagic organism decline” attributed to flow alteration and declining food supplies in the SF Estuary (MacNally et al., 2010; Sommer et al., 2007). While negative covariance between estuarine dependent and marine opportunist life histories occurred in recent years, the persistently low aggregated mean for anadromous species signals a weakened portfolio for that life history. Overall, the results of this study support the idea that the preservation of life history diversity based on salinity guilds (e.g., oligohaline, mesohaline, polyhaline; Feyrer et al., 2015), thermal guilds (e.g., cool‐tolerant vs. warm‐tolerant), and migration patterns (e.g., resident vs. migratory) is required to increase biological insurance among the portfolio (Anderson et al., 2015).

### 4.3 Climate threats to biodiversity, food webs, and ecosystems

Juvenile fish species that respond synchronously to regionally coordinated environmental drivers may be at heightened risk of multiple consecutive recruitment failures due to climate change, as recently described from other groups and ecosystems (Arimitsu et al., 2021; Kahilainen et al., 2018; Sarremejane et al., 2021). In our study, juveniles of marine opportunist and estuarine‐dependent species that typically enter the SF estuary during cooler SSTs may be at increased risk of recruitment failures during marine heatwaves, which are expected to intensify in frequency, magnitude, and duration off the California coast (Sanford et al., 2019). Though these cool‐tolerant marine fishes may be “losers” in the SF Estuary under climate warming, they may be able to gradually expand their distributions northward to more suitable estuaries of the Northeast Pacific (Cheung et al., 2015). Anadromous species may be at the highest risk of losing because freshwater flow variability governs the conditions that they encounter during rearing and outmigration (Hobbs et al., 2010; Kimmerer et al., 2013; Meng & Matern, 2001). Longfin Smelt is particularly at high risk of local extirpation in the SF Estuary, at the southern edge of its range, due to its short life cycle (1–3 years) and dependence on high flow years for successful recruitment (Moyle, 2002). Chinook Salmon, an anadromous and historically abundant fish, although not captured frequently enough by the SF Bay Study to be included in this analysis, has also declined to low levels and shows increased population synchrony through time (Carlson & Satterthwaite, 2011). Importantly, spatial synchrony in California salmonids is expected to be further exacerbated by climate change (Katz et al., 2013; Moyle et al., 2013). It remains unclear the extent to which anadromous species could overcome multiple consecutive recruitment failures during prolonged drought by producing strong year classes during flood years—in response to the “weather whiplash” scenario that has become frequent over the past two decades and is projected to intensify over this century (Swain et al., 2018). Future climate variability and trends may lead to a juvenile fish community dominated by “winners” that have high physiological tolerances, can adapt rapidly in response to the shifting conditions, and can opportunistically take advantage of spatially limited or infrequent occurrences of favorable conditions to produce strong year classes (Moyle et al., 2013). Endemic species may face extinction if early life stages cannot tolerate the cumulative impacts of multiple stressors in the estuary (e.g., the Delta Smelt *Hypomesus transpacificus*; Hobbs et al., 2017).

Climate change impacts on the diversity of juvenile fishes may alter energy flows through food webs by narrowing the quantity and/or quality of nutrients and energy available to higher‐level consumers (Santora et al., 2021; Thayer et al., 2014; Thompson, Harvey, et al., 2019). In this study, we found no significant effects of SSTs on Northern Anchovy in the SF Estuary despite its documented association of increased abundance during cool SSTs (Chavez et al., 2003). Instead, we observed high abundances of Northern Anchovy in Central Bay during the Pacific marine heatwave of 2014–2016, which aligns with observations from ocean monitoring data (Thompson, Schroeder, et al., 2019). During this warm water anomaly, coastal predator populations collapsed, likely due to a narrowing of forage fish diversity dominated by Northern Anchovy, which has high concentrations of thiaminase, an enzyme that breaks down thiamine and thus affects nerve, muscle, and heart function (Santora et al., 2021). Currently, researchers are investigating the role of thiamine deficiency in the observed high offspring mortality of adult female Chinook Salmon that were foraging in the ocean during marine heatwaves and then returned to rivers to spawn (Mantua et al., 2021). Dedicated studies linking climate‐induced fluctuations in juvenile fish production in estuaries to higher‐level consumers are urgently needed to understand how climate change may affect not only species but also food webs and ecosystems across the marine‐freshwater gradient.

### 4.4 Conservation management implications

Balancing the opposing needs of freshwater and marine species that respond strongly to environmental conditions requires special consideration from managers. During prolonged drought, periodic managed flow pulses that freshen the Delta and downstream regions during the rearing and outmigration windows could strengthen cohorts of anadromous species (e.g., similar to Chinook Salmon; Munsch et al., 2019), and even estuarine dependent ones (e.g., Starry Flounder, Bay Goby, Pacific Staghorn Sculpin). In contrast, the periodic expansion of saltier upstream habitat during drought may continue to benefit marine species such as Pacific Herring, Northern Anchovy, and English Sole, depending on prevailing SSTs. Managing flow pulses to shorten summer peak temperatures (Cloern et al., 2011) and restoring deep bays and shallow tidal marshes that encourage thermal stratification and evaporative cooling (Enright et al., 2013; Vroom et al., 2017) could help mitigate marine heatwave impacts. Regionally coordinated efforts to prevent consecutive recruitment failures for high‐risk species, particularly anadromous species, is critical for their persistence in the SF Estuary.

### 4.5 Advances in time series modeling, caveats, and recommended future directions

Our work leverages advances in multivariate time series modeling, and benefits from the increasing availability of long‐term, spatially replicated, publicly available monitoring data sets (sometimes referred to as a “big data revolution” in ecology; Hampton et al., 2013). However, limitations to our analysis warrant further discussion. Our broad‐scale analysis of population dynamics evaluates patterns at annual scales, across a longitudinal marine‐freshwater gradient, and in response to freshwater flows and SSTs. While we found that flow and SST can act as synchronizing drivers of abundance fluctuations, positive spatial covariance among species and regions suggests that spatial synchrony may also be driven by other environmental effects and/or non‐environmental effects (e.g., dispersal, food availability, competition, predator–prey interactions; Ims & Steen, 1990, Leibold et al., 2004, Walter et al., 2021). Further consideration of environmental drivers, fish behavior, and resource use could help explain this spatial covariance.

Future directions are to examine fine‐scale habitat and environmental relationships at sub‐annual scales using abundance, movement, or geochemical data (see Colombano et al., 2020; Hobbs et al., 2019; Stowell et al., 2019; Sturrock et al., 2020). Additionally, although we discuss our findings in the context of documented regime shifts in the ocean and estuary, here we only tested for the effects of the *Potamocorbula amurensis* invasion of 1987 (see Table S2; Cloern & Jassby, 2012; Sommer et al., 2007; Winder & Jassby, 2011). Hindcast‐forecast approaches using downscaled climate models and flow scenarios (e.g., Knowles & Cronkite‐Ratcliff, 2018) could be applied to better detect change points in environmental conditions, other potential regime shifts (Wilson et al., 2021), and associated quasi‐extinction risks in the fish community (sensu Ruhi et al., 2016; Sarremejane et al., 2021). Despite these limitations, our data‐driven analysis improves on previous efforts to quantify climate‐fish abundance relationships across the estuary by incorporating state and observation processes and by combining data from multiple gears. Our approach is transferable to other systems with spatially replicated, long‐term data on fish abundance, distribution, and environmental drivers, and could help understand climate change impacts on fish communities spanning marine‐freshwater gradients worldwide.

## 5 CONCLUSION

Across the globe, climate change is altering physical processes in estuaries and coasts, which may ultimately diminish their capacity to function as nurseries for a wide variety of economically, recreationally, and ecologically significant fish species (Lauchlan & Nagelkerken, 2020). Species with early life stages that are maladapted to novel conditions in the estuary may either shift their distributions to more suitable climates or become locally imperiled due to repeated recruitment failures. Preventing species losses in the SF Estuary and other river‐dominated estuaries may require preserving and restoring biocomplexity: dynamic flow regimes, heterogeneous salinity gradients, thermal refugia, and habitat mosaics that collectively maintain or even increase the spatial and temporal availability of favorable conditions for species with different life histories (Broadley et al., 2022; Colombano et al., 2020; Moyle et al., 2010). Long‐term monitoring programs will be increasingly critical to understand the capacity of estuaries to consistently function as nursery areas for broad suites of fishes under accelerating climate change.

## Supporting information


Appendix S1.

## Data Availability

The data, metadata, and scripts that support the findings of this study are archived in a Dryad repository (https://doi.org/10.6078/D19T3G). These data were derived from the following resources available in the public domain: (1) Department of California Fish and Wildlife's San Francisco Bay Study (https://filelib.wildlife.ca.gov/Public/BayStudy/); (2) UCSD NOAA Shore Stations Farallon Islands station (https://shorestations.ucsd.edu/data‐farallon/); and (3) California Department of Water Resources dayflow (https://data.cnra.ca.gov/dataset/dayflow).
