# Supplementary material for: Four decades of climatic fluctuations and fish recruitment stability across a marine‐freshwater gradient
Source: Glob Chang Biol. 2022 Jun 16;28(17):5104–20. doi: 10.1111/gcb.16266 (PMC9545339; doi:10.1111/gcb.16266)
Supplement: Supplementary file 1 — Appendix S1. [file GCB-28-5104-s001.pdf]

Supplementary material

Table S1. Mean +/- standard deviation (SD) of salinity (PSU) and temperature (°C) associated with each age-0 fish species sampled during the SF Bay Study from April to October 1980 to 2018. Bottom measurements correspond to otter trawls (OT); surface measurements correspond to midwater trawls (MWT); and average measurements represent conditions along the entire water column, corresponding to species captured by both gear types. Missing data points were removed from calculations.

Species (Age 0)	Sample	Salinity (PSU): mean (SD)	Temperature (°C): mean (SD)
Longfin Smelt	Average	14.51 (9.23)	18.45 (1.98)
Striped Bass	Average	7.64 (6.64)	19.17 (2.46)
American Shad	Surface	10.94 (9.15)	19.69 (1.71)
California Tonguefish	Bottom	27.28 (4.60)	17.75 (2.29)
Brown Rockfish	Bottom	28.88 (3.65)	17.27 (1.99)
Speckled Sanddab	Bottom	27.16 (4.94)	16.39 (2.21)
English Sole	Bottom	25.97 (5.48)	16.26 (2.03)
Northern Anchovy	Surface	23.44 (7.53)	18.02 (2.21)
White Croaker	Bottom	26.81 (5.42)	16.75 (2.06)
Yellowfin Goby	Bottom	14.55 (10.38)	19.10 (1.76)
Bay Goby	Bottom	24.59 (6.41)	16.30 (2.10)
Shiner Surfperch	Average	27.85 (4.27)	18.44 (1.54)
Starry Flounder	Bottom	10.99 (8.55)	19.51 (1.48)
Pacific Herring	Surface	22.56 (8.24)	16.96 (2.06)
Plainfin Midshipman	Bottom	27.47 (5.84)	18.42 (1.85)
Jacksmelt	Surface	28.30 (3.92)	18.43 (1.71)
Pacific Staghorn Sculpin	Bottom	20.82 (9.73)	17.39 (2.19)
Walleye Surfperch	Surface	28.06 (4.04)	17.90 (1.42)

Table S2. Model comparisons for all age-0 species and covariate combinations. Model covariates abbreviations are freshwater flow (Flow), sea surface temperature (SST), residual spatial covariance (SpCov), Secchi in the observation model (Secchi), and a dummy variable for the regime shift attributed to the *Potamocorbula amurensis* invasion of 1987 (Clam). Other model parameters are the log likelihood (logLik), corrected Akaike Information Criterion (AICc), number of parameters (num.param), number of iterations (num.iter), whether the model converged (TRUE/FALSE), the difference in AICc from the top-ranked model (delta.AICc), the relative likelihood (rel.like), and the relative model weight assigned to each model (AIC.wt; 0-1).

Species (Age 0)	Model	logLik	AICc	num. param	num. iter	con-verged	delta. AICc	rel.like	AIC.wt
LONSME	SpCov + Flow	-1826	3727	36	249	TRUE	0	1	0.86
LONSME	SpCov + Flow + SST + Secchi	-1821	3732	44	242	TRUE	5	0	0.07
LONSME	SpCov	-1834	3733	32	247	TRUE	6	0	0.04
LONSME	SpCov + Flow + SST	-1825	3734	40	247	TRUE	7	0	0.03
LONSME	SpCov + SST	-1832	3738	36	247	TRUE	11	0	0.00
LONSME	SpCov + Flow + SST + Clam	-1824	3739	44	241	TRUE	12	0	0.00
LONSME	Null	-1899	3851	26	967	TRUE	124	0	0.00
STRBAS	SpCov + Flow	-1510	3076	27	577	TRUE	0	1	0.50
STRBAS	SpCov + Flow + SST	-1507	3077	30	351	TRUE	0	1	0.42
STRBAS	SpCov + Flow + SST + Secchi	-1507	3082	33	344	TRUE	6	0	0.03
STRBAS	SpCov + Flow + SST + Clam	-1507	3082	33	351	TRUE	6	0	0.03
STRBAS	SpCov	-1517	3084	24	435	TRUE	8	0	0.01
STRBAS	SpCov + SST	-1514	3084	27	382	TRUE	8	0	0.01
STRBAS	Null	-1548	3138	21	43	TRUE	62	0	0.00
AMESHA	SpCov + Flow	-572	1188	21	220	TRUE	0	1	0.59
AMESHA	SpCov	-576	1190	18	220	TRUE	2	0	0.27
AMESHA	SpCov + Flow + SST + Clam	-568	1192	27	322	TRUE	4	0	0.09
AMESHA	SpCov + Flow + SST	-572	1195	24	261	TRUE	6	0	0.03
AMESHA	SpCov + SST	-576	1196	21	221	TRUE	8	0	0.01
AMESHA	SpCov + Flow + SST + Secchi	-571	1198	27	223	TRUE	9	0	0.01
AMESHA	Null	-623	1276	15	473	TRUE	88	0	0.00
CALTON	SpCov + Flow + SST + Clam	-457	951	17	249	TRUE	0	1	0.45
CALTON	SpCov + Flow + SST + Secchi	-457	951	17	303	TRUE	0	1	0.43
CALTON	SpCov + Flow	-463	954	13	68	TRUE	4	0	0.08
CALTON	SpCov	-467	957	11	26	TRUE	6	0	0.02
CALTON	SpCov + Flow + SST	-463	957	15	74	TRUE	7	0	0.02
CALTON	SpCov + SST	-466	960	13	26	TRUE	10	0	0.00
CALTON	Null	-501	1022	10	15	TRUE	71	0	0.00
BROROC	SpCov + SST	-127	286	13	279	TRUE	0	1	0.54
BROROC	SpCov	-131	287	11	326	TRUE	1	0	0.27
BROROC	SpCov + Flow	-129	289	13	291	TRUE	3	0	0.11
BROROC	SpCov + Flow + SST	-126	290	15	264	TRUE	4	0	0.06
BROROC	SpCov + Flow + SST + Clam	-125	294	17	246	TRUE	9	0	0.01
BROROC	SpCov + Flow + SST + Secchi	-125	295	17	261	TRUE	9	0	0.01
BROROC	Null	-139	302	10	73	TRUE	16	0	0.00
SPESAN	SpCov + Flow + SST + Secchi	-1346	2749	27	239	TRUE	0	1	1.00
SPESAN	SpCov + SST	-1360	2763	21	273	TRUE	14	0	0.00
SPESAN	SpCov + Flow + SST	-1358	2765	24	273	TRUE	17	0	0.00
SPESAN	SpCov	-1365	2767	18	475	TRUE	19	0	0.00
SPESAN	SpCov + Flow	-1362	2768	21	424	TRUE	19	0	0.00
SPESAN	SpCov + Flow + SST + Clam	-1356	2768	27	255	TRUE	20	0	0.00
SPESAN	Null	-1401	2833	15	16	TRUE	84	0	0.00
ENGSOL	SpCov + Flow + SST	-1715	3479	24	229	TRUE	0	1	0.59
ENGSOL	SpCov + Flow + SST + Secchi	-1712	3480	27	229	TRUE	1	1	0.35
ENGSOL	SpCov + Flow + SST + Clam	-1714	3484	27	223	TRUE	5	0	0.04
ENGSOL	SpCov + Flow	-1721	3486	21	261	TRUE	7	0	0.02
ENGSOL	SpCov + SST	-1725	3494	21	297	TRUE	15	0	0.00
ENGSOL	SpCov	-1731	3499	18	284	TRUE	20	0	0.00
ENGSOL	Null	-1756	3543	15	18	TRUE	64	0	0.00
NORANC	SpCov	-1866	3785	26	434	TRUE	0	1	0.51

NORANC	SpCov + Flow	-1862	3785	30	461	TRUE	0	1	0.43
NORANC	SpCov + Flow + SST	-1861	3791	34	517	TRUE	6	0	0.02
NORANC	SpCov + Flow + SST + Clam	-1861	3791	34	517	TRUE	6	0	0.02
NORANC	SpCov + SST	-1865	3792	30	481	TRUE	8	0	0.01
NORANC	SpCov + Flow + SST + Secchi	-1859	3796	38	492	TRUE	11	0	0.00
NORANC	Null	-1880	3801	20	20	TRUE	17	0	0.00
WHICRO	SpCov + Flow + SST + Clam	-1060	2176	27	348	TRUE	0	1	0.75
WHICRO	SpCov + Flow + SST + Secchi	-1062	2180	27	5000	FALSE	4	0	0.12
WHICRO	SpCov	-1072	2181	18	246	TRUE	5	0	0.08
WHICRO	SpCov + SST	-1069	2183	21	341	TRUE	6	0	0.03
WHICRO	SpCov + Flow	-1070	2184	21	235	TRUE	8	0	0.02
WHICRO	SpCov + Flow + SST	-1068	2186	24	4730	TRUE	10	0	0.01
WHICRO	Null	-1097	2225	15	25	TRUE	49	0	0.00
YELGOB	SpCov	-991	2020	18	230	TRUE	0	1	0.77
YELGOB	SpCov + Flow	-990	2023	21	230	TRUE	3	0	0.15
YELGOB	SpCov + SST	-991	2025	21	233	TRUE	5	0	0.07
YELGOB	SpCov + Flow + SST	-989	2028	24	233	TRUE	9	0	0.01
YELGOB	SpCov + Flow + SST + Secchi	-989	2034	27	244	TRUE	14	0	0.00
YELGOB	SpCov + Flow + SST + Clam	-989	2035	27	234	TRUE	15	0	0.00
YELGOB	Null	-1057	2145	15	15	TRUE	125	0	0.00
BAYGOB	SpCov + Flow + SST + Secchi	-1483	3023	27	229	TRUE	0	1	0.93
BAYGOB	SpCov + SST	-1493	3029	21	289	TRUE	7	0	0.03
BAYGOB	SpCov + Flow + SST + Clam	-1487	3030	27	384	TRUE	7	0	0.02
BAYGOB	SpCov + Flow + SST	-1491	3030	24	235	TRUE	8	0	0.02
BAYGOB	SpCov	-1499	3035	18	241	TRUE	12	0	0.00
BAYGOB	SpCov + Flow	-1498	3039	21	237	TRUE	16	0	0.00
BAYGOB	Null	-1552	3134	15	15	TRUE	111	0	0.00
SHIPER	SpCov + SST	-999	2050	25	350	TRUE	0	1	0.81
SHIPER	SpCov + Flow + SST	-998	2055	28	370	TRUE	4	0	0.09
SHIPER	SpCov	-1005	2056	22	256	TRUE	5	0	0.06
SHIPER	SpCov + Flow	-1003	2058	25	257	TRUE	8	0	0.02
SHIPER	SpCov + Flow + SST + Secchi	-997	2059	31	414	TRUE	9	0	0.01
SHIPER	SpCov + Flow + SST + Clam	-997	2060	31	461	TRUE	10	0	0.01
SHIPER	Null	-1016	2070	19	68	TRUE	20	0	0.00
STAFLO	SpCov	-516	1069	18	245	TRUE	0	1	0.54
STAFLO	SpCov + Flow	-513	1070	21	246	TRUE	1	1	0.32
STAFLO	SpCov + SST	-514	1073	21	237	TRUE	3	0	0.10
STAFLO	SpCov + Flow + SST	-512	1075	24	238	TRUE	6	0	0.03
STAFLO	SpCov + Flow + SST + Secchi	-509	1077	27	561	TRUE	8	0	0.01
STAFLO	SpCov + Flow + SST + Clam	-511	1081	27	240	TRUE	12	0	0.00
STAFLO	Null	-547	1126	15	54	TRUE	57	0	0.00
PACHER	SpCov + Flow	-1658	3377	30	265	TRUE	0	1	0.38
PACHER	SpCov + Flow + SST	-1654	3378	34	391	TRUE	1	1	0.22
PACHER	SpCov	-1663	3378	26	267	TRUE	1	1	0.20
PACHER	SpCov + SST	-1658	3379	30	394	TRUE	1	0	0.18
PACHER	SpCov + Flow + SST + Secchi	-1653	3385	38	416	TRUE	8	0	0.01
PACHER	SpCov + Flow + SST + Clam	-1653	3386	38	393	TRUE	9	0	0.01
PACHER	Null	-1703	3447	20	24	TRUE	70	0	0.00
PLAMID	SpCov + Flow + SST + Secchi	-1595	3270	38	306	TRUE	0	1	1.00
PLAMID	SpCov + Flow	-1613	3288	30	364	TRUE	19	0	0.00
PLAMID	SpCov + Flow + SST	-1609	3288	34	388	TRUE	19	0	0.00
PLAMID	SpCov + SST	-1614	3290	30	330	TRUE	20	0	0.00
PLAMID	SpCov	-1619	3292	26	366	TRUE	22	0	0.00
PLAMID	SpCov + Flow + SST + Clam	-1608	3295	38	5000	FALSE	25	0	0.00
PLAMID	Null	-1639	3320	20	613	TRUE	50	0	0.00
JACKSM	SpCov + Flow + SST + Clam	-964	1984	27	303	TRUE	0	1	0.33
JACKSM	SpCov + Flow	-971	1984	21	423	TRUE	0	1	0.30
JACKSM	SpCov + SST	-971	1985	21	495	TRUE	1	1	0.22
JACKSM	SpCov + Flow + SST	-969	1987	24	544	TRUE	3	0	0.08
JACKSM	SpCov + Flow + SST + Secchi	-966	1987	27	221	TRUE	3	0	0.07
JACKSM	Null	-1002	2034	15	92	TRUE	50	0	0.00
PACSSC	SpCov	-2058	4188	35	390	TRUE	0	1	0.55
PACSSC	SpCov + SST	-2054	4190	40	370	TRUE	2	0	0.22

PACSSC	SpCov + Flow	-2054	4191	40	452	TRUE	3	0	0.12
PACSSC	SpCov + Flow + SST	-2049	4192	45	380	TRUE	4	0	0.08
PACSSC	SpCov + Flow + SST + Secchi	-2045	4194	50	403	TRUE	6	0	0.03
PACSSC	SpCov + Flow + SST + Clam	-2048	4200	50	374	TRUE	12	0	0.00
PACSSC	Null	-2124	4298	25	782	TRUE	110	0	0.00
WALSUR	Null	-49.6	111	5	69	TRUE	0	1	0.30
WALSUR	SpCov	-49.6	111	5	69	TRUE	0	1	0.30
WALSUR	SpCov + Flow	-48.6	112	6	2201	TRUE	1	1	0.21
WALSUR	SpCov + SST	-48.9	113	6	41	TRUE	1	1	0.15
WALSUR	SpCov + Flow + SST	-48.8	115	7	47	TRUE	4	0	0.04
WALSUR	SpCov + Flow + SST + Clam	-48.4	118	8	67	TRUE	7	0	0.01

Table S3. Theil-Sen robust estimates for each ‘state’ (i.e., observation error free abundance estimates) generated by the MARSS models, based on the “Full” model. Estuary-wide represents the TS estimate for average abundance across the estuary. Significance codes: 0 ‘***’ 0.001 ‘**’ 0.01 ‘*’ 0.05.

Species (Age 0)	Region	Theil-Sen (TS)	P-value	Signif. code
Longfin Smelt	Estuary-wide	-0.1232	3.29E-09	***
Longfin Smelt	West Delta	-0.04953	7.28E-12	***
Longfin Smelt	Suisun Bay	-0.10379	7.28E-12	***
Longfin Smelt	San Pablo Bay	-0.1257	1.25E-05	***
Longfin Smelt	Central Bay	-0.2537	6.94E-07	***
Striped Bass	Estuary-wide	-0.14756	3.64E-12	***
Striped Bass	West Delta	-0.14751	7.28E-12	***
Striped Bass	Suisun Bay	-0.1844	3.64E-12	***
Striped Bass	San Pablo Bay	-0.11552	3.64E-12	***
American Shad	Estuary-wide	0.01882	0.0249	*
American Shad	West Delta	0.02335	0.0600	
American Shad	Suisun Bay	0.01339	0.0222	*
American Shad	San Pablo Bay	0.02051	0.00224	**
California Tonguefish	Estuary-wide	-0.02552	0.283	
California Tonguefish	Central Bay	-0.01686	0.576	
California Tonguefish	South Bay	-0.02484	0.674	
Brown Rockfish	Estuary-wide	0.1826	1.95E-09	***
Brown Rockfish	Central Bay	0.2423	2.09E-08	***
Brown Rockfish	South Bay	0.0709	8.64E-09	***
Speckled Sanddab	Estuary-wide	1.1145	3.64E-12	***
Speckled Sanddab	San Pablo Bay	0.1555	0.00118	***
Speckled Sanddab	Central Bay	2.9194	3.64E-12	***
Speckled Sanddab	South Bay	0.09149	0.0213	*
English Sole	Estuary-wide	1.3562	2.55E-11	***
English Sole	San Pablo Bay	0.8952	2.09E-08	***
English Sole	Central Bay	3.178	7.28E-12	***
English Sole	South Bay	0.1592	0.000219	***
Northern Anchovy	Estuary-wide	-3.330	0.000138	***
Northern Anchovy	Suisun Bay	-0.05051	0.0414	*
Northern Anchovy	San Pablo Bay	-2.866	0.000129	***
Northern Anchovy	Central Bay	-15.17	9.75E-05	***
Northern Anchovy	South Bay	1.998	0.00172	**
White Croaker	Estuary-wide	-0.4954	5.09E-11	***
White Croaker	San Pablo Bay	-0.2619	7.28E-12	***
White Croaker	Central Bay	-0.4558	1.35E-09	***
White Croaker	South Bay	-0.4786	1.56E-10	***
Yellowfin Goby	Estuary-wide	-0.2417	1.17E-08	***
Yellowfin Goby	West Delta	-0.1779	1.35E-09	***
Yellowfin Goby	Suisun Bay	-0.3754	1.95E-09	***
Yellowfin Goby	San Pablo Bay	-0.2192	1.56E-06	***
Bay Goby	Estuary-wide	0.4430	0.000343	***
Bay Goby	San Pablo Bay	0.5698	3.01E-05	***
Bay Goby	Central Bay	0.4322	9.75E-05	***
Bay Goby	South Bay	0.3406	0.00519	**
Shiner Surfperch	Estuary-wide	-0.2516	3.64E-12	***
Shiner Surfperch	San Pablo Bay	-0.2270	3.64E-12	***
Shiner Surfperch	Central Bay	-0.2583	0.000749	***
Shiner Surfperch	South Bay	-0.2494	3.64E-12	***
Starry Flounder	Estuary-wide	-0.0863	0.0149	*

Starry Flounder	West Delta	0.02935	0.150	
Starry Flounder	Suisun Bay	0.0314	0.530	
Starry Flounder	San Pablo Bay	-0.1476	0.00139	**
Pacific Herring	Estuary-wide	-0.0852	0.253	
Pacific Herring	Suisun Bay	-0.003823	0.162	
Pacific Herring	San Pablo Bay	-0.1183	0.0386	*
Pacific Herring	Central Bay	-0.1218	0.556	
Pacific Herring	South Bay	-0.05643	8.47E-05	***
Plainfin Midshipman	Estuary-wide	1.0627	1.12E-09	***
Plainfin Midshipman	Suisun Bay	0.09068	0.000138	***
Plainfin Midshipman	San Pablo Bay	0.2207	1.72E-06	***
Plainfin Midshipman	Central Bay	3.117	3.29E-09	***
Plainfin Midshipman	South Bay	0.4520	1.56E-10	***
Jacksmelt	Estuary-wide	0.00222	0.735	
Jacksmelt	San Pablo Bay	-0.01862	0.0057	**
Jacksmelt	Central Bay	0.02802	0.0175	*
Jacksmelt	South Bay	0.005231	0.863	
Pacific Staghorn Sculpin	Estuary-wide	0.0541	0.395	
Pacific Staghorn Sculpin	West Delta	-0.01555	0.0798	
Pacific Staghorn Sculpin	Suisun Bay	0.01807	0.831	
Pacific Staghorn Sculpin	San Pablo Bay	-0.3469	0.00213	**
Pacific Staghorn Sculpin	Central Bay	0.5122	2.30E-06	***
Pacific Staghorn Sculpin	South Bay	0.05102	0.0300	*
Walleye Surfperch	Estuary-wide	0.06432	0.000129	***
Walleye Surfperch	Central Bay	0.06432	0.000129	***

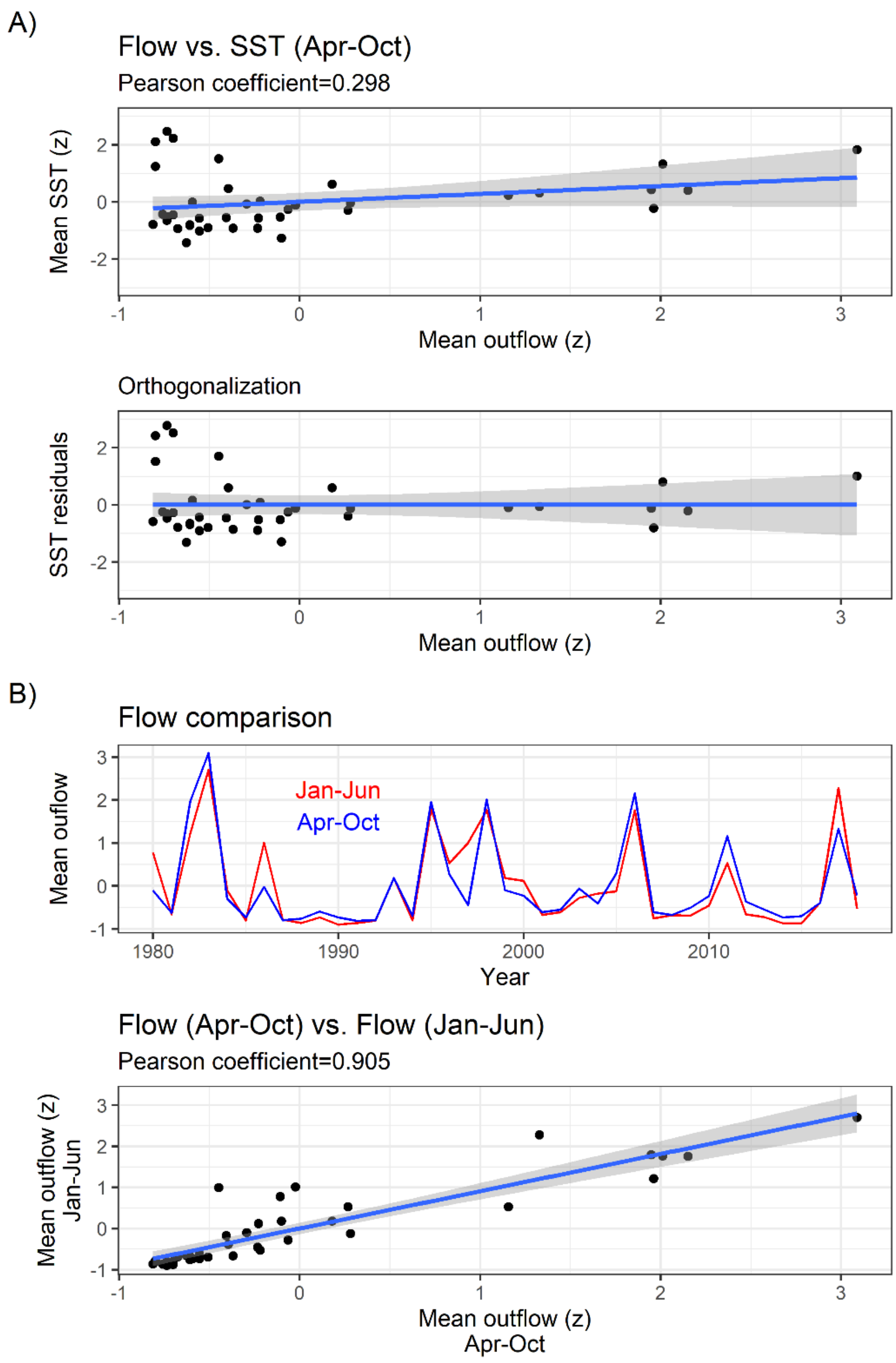

Figure S1. Pre-screening covariates for MARSS models. (A) Multicollinearity among covariates used in MARSS models was assessed using Pearson's correlation test and orthogonalization of residuals. (B) Mean annual net Delta outflow from April to October was compared to January to June 1980 to 2018. High correlation is interpreted as the April to October window, which matches the fish sampling window, being suitable for capturing annual trends.

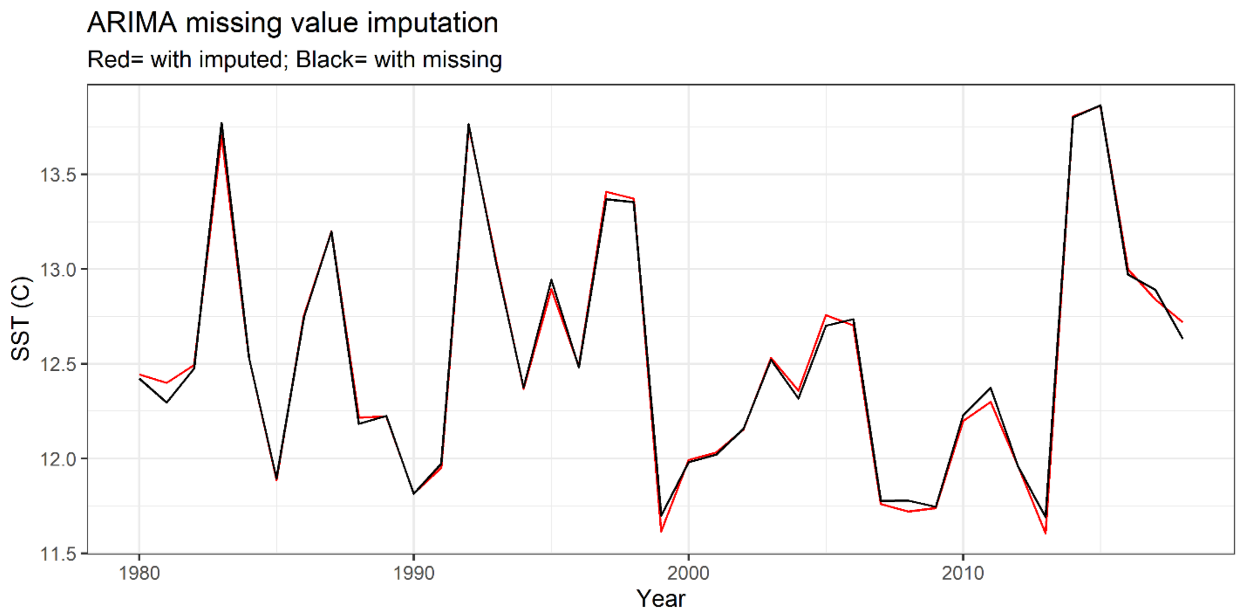

Figure S2. UCSD **/** NOAA Shore Stations – Farallon Island Station SST (°C) time series with and without missing values. In the dataset from 1980-2018, 1,837 daily values were either missing (NA) or flagged (uncertain) of 14,245 total values. Missing values were imputed with a seasonal autoregressive integrated moving average (ARIMA) model before being summarized into annual means from April to October 1980 to 2018, screened for collinearity, and included in MARSS models. See Methods for details.

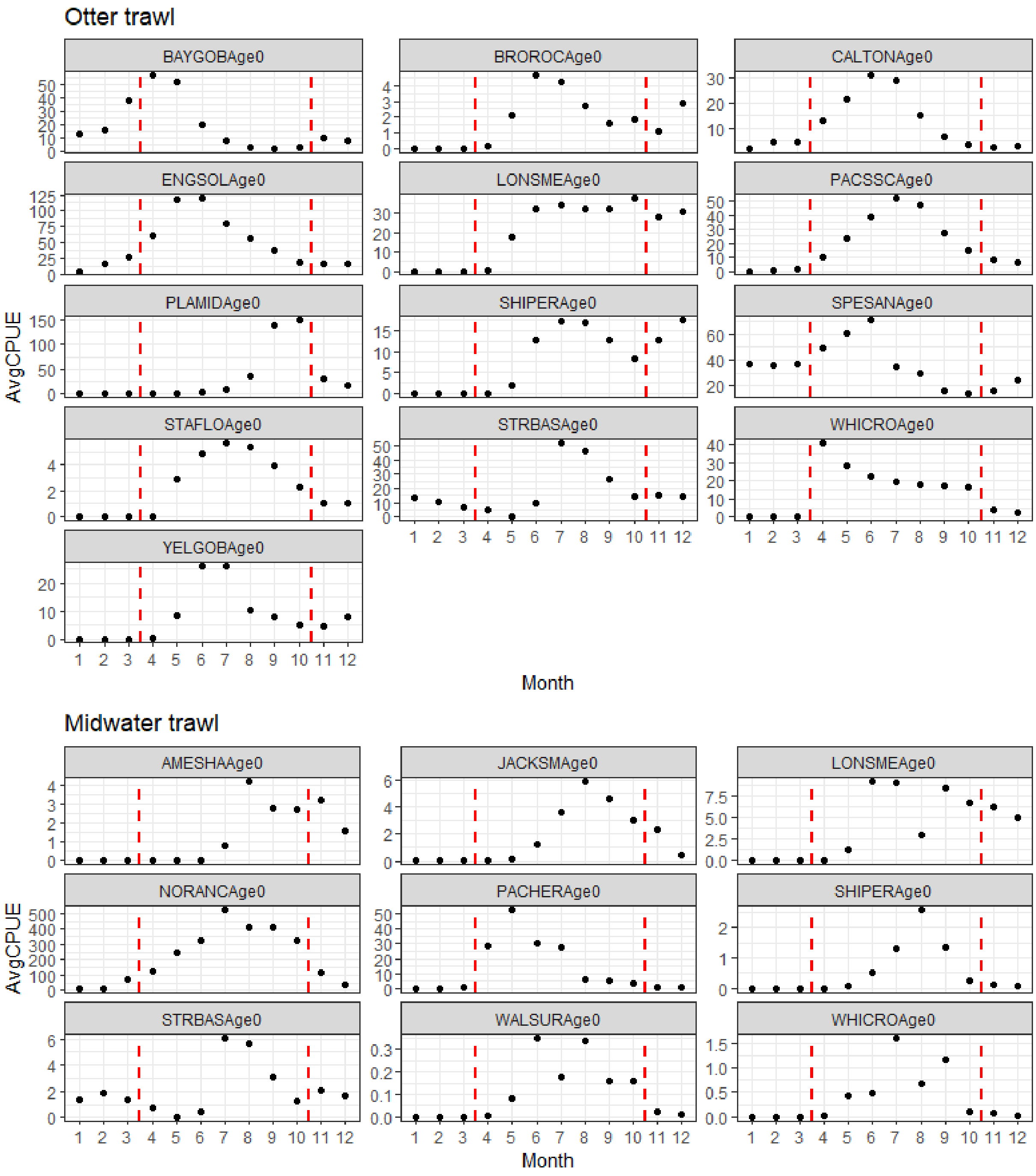

Figure S3. Seasonal mean CPUE of age-0 fishes in the raw CDFW SF Bay Study dataset (1980-2018). Selection of the months April to October (indicated with red dashed vertical lines), when sampling density was most consistent, captures peak age-0 fish catches.

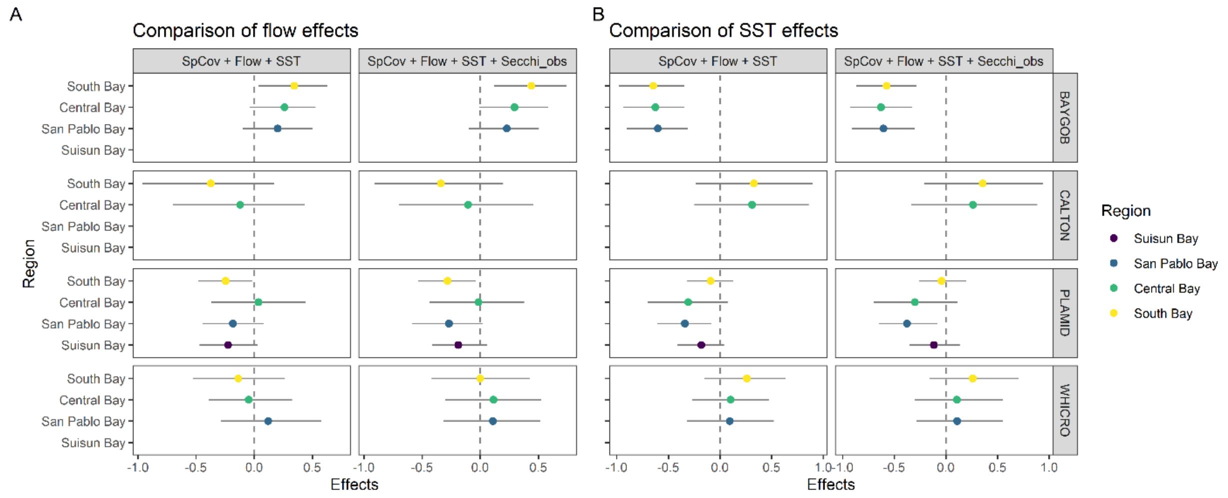

Figure S4. Effect size comparison for (A) freshwater flow and (B) sea surface temperature (SST) effects on age-0 fish abundance for four species where the inclusion of Secchi in the observation model ("Secchi") improved AICc scores >2 units. The panels show covariate effects according to two model specifications: (Left) SpCov + Flow + SST; (Right) SpCov + Flow + SST + Secchi in the observation model. See Methods, and Table S1 for more details.

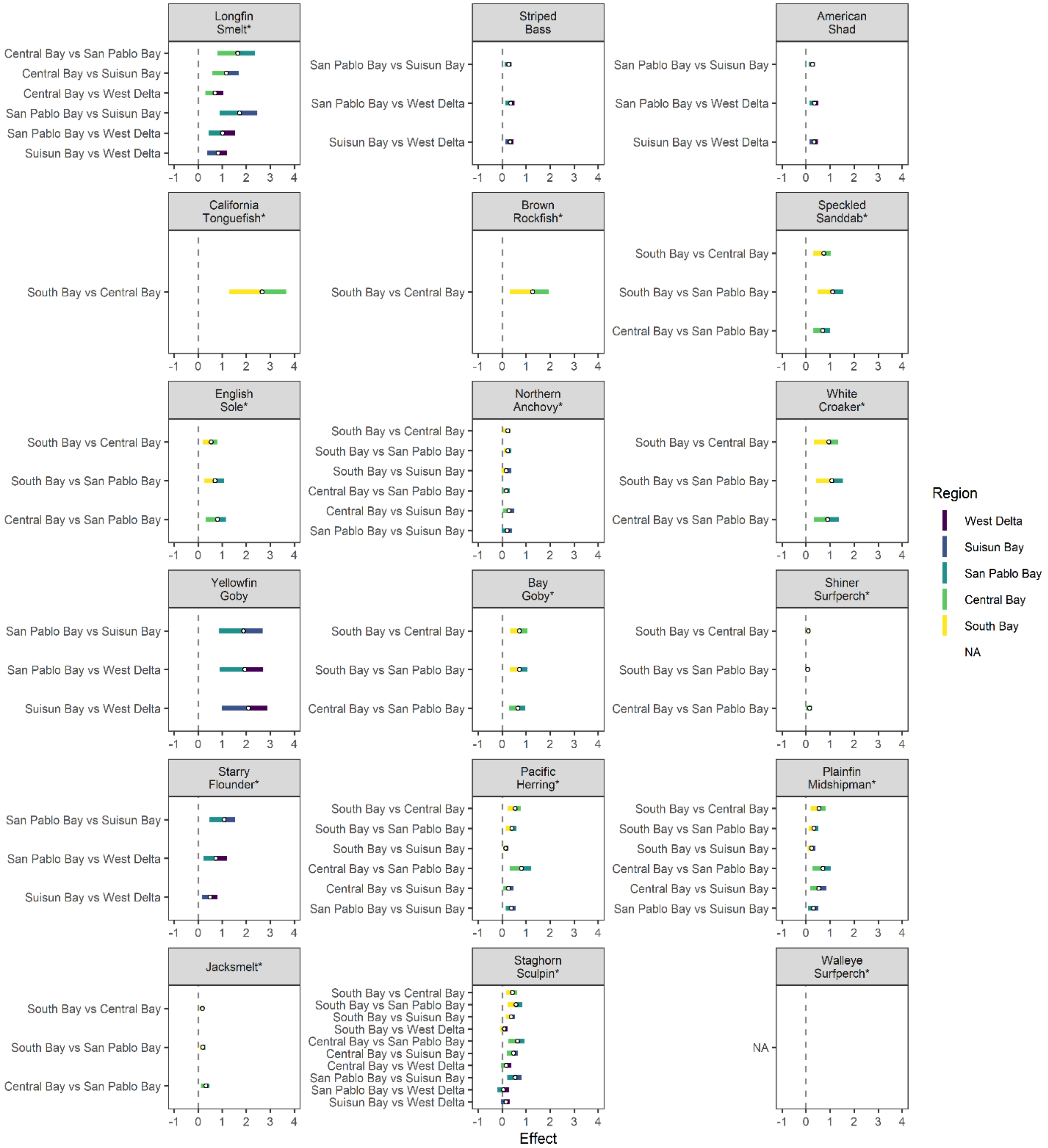

Figure S5. MARSS "full" model (SpCov + Flow + SST) estimates of process error covariance ('Q' parameter estimated with the "unconstrained" setting; interpreted as residual spatial covariance between two regions) after accounting for the effects of environmental covariates.

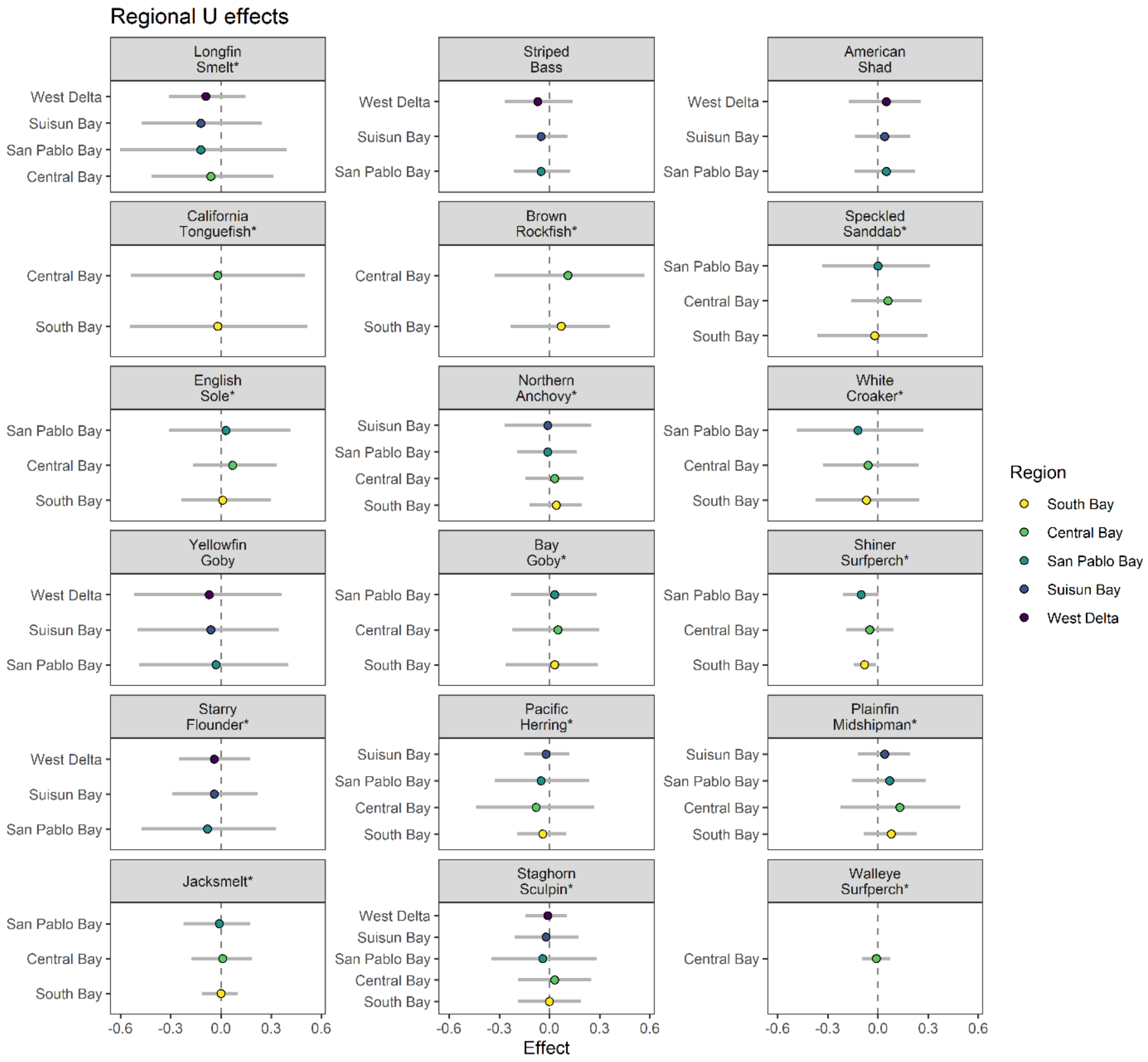

Figure S6. MARSS “full” model (SpCov + Flow + SST) estimates of intrinsic growth (‘U’ parameter; interpreted as long-term trend after accounting for environmental covariates).

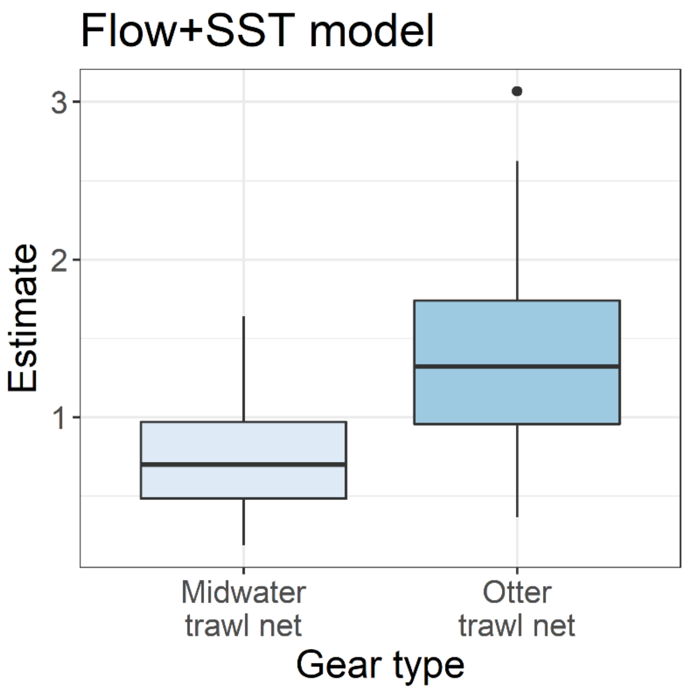

Figure S7. MARSS “full” model (SpCov + Flow + SST) estimates of observation error variance (‘R’ parameter) pooled across age-0 species and gear types.

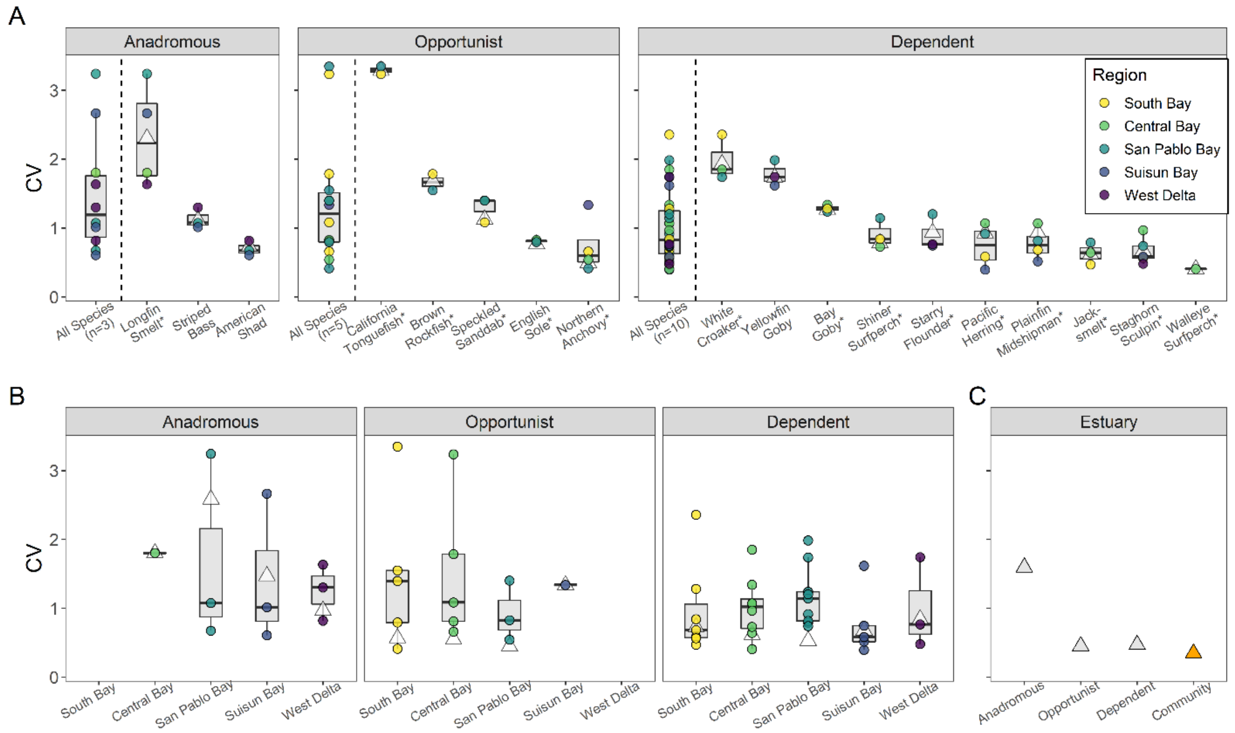

Figure S8. Portfolio effects confer juvenile recruitment stability: a stepwise dampening of interannual variability emerges as biological and spatial scales increase, whereby lower coefficient of variation (CV) values are interpreted as more stable. (A) Species (region) variability is shown as color-coded circles with box and whisker plots representing the mean and interquartile range (IQR) among regions. Species (estuary) variability is shown as white triangles. (B) Life history (region) variability is shown as color-coded circles with box and whisker plots representing the mean and IQR for each region. Life history (estuary) variability is shown as white triangles. (C) At the estuary-wide scale, life history variability is shown as grey triangles and community variability is shown as an orange triangle.
